# Drug administration via feeding tubes—a procedure that carries risks: systematic identification of critical factors based on commonly administered drugs in a cohort of stroke patients

**DOI:** 10.1007/s00228-024-03723-4

**Published:** 2024-07-29

**Authors:** Jana Sommerfeldt, Hannes Sartorius, Bettina von Sarnowski, Sandra Klein, Christoph A. Ritter

**Affiliations:** 1 University of Greifswald, Institute of Pharmacy, Clinical Pharmacy, Greifswald, Germany; 2 University Medicine Greifswald, Department of Neurology, Greifswald, Germany; 3 University of Greifswald, Institute of Pharmacy, Biopharmaceutics and Pharmaceutical Technology, Greifswald, Germany

**Keywords:** Drug administration, Feeding tube, Critical factors, Stroke patients

## Abstract

**Purpose:**

Drug administration via feeding tubes is considered a process with many uncertainties. This review aimed to give a comprehensive overview of data available on feeding tube application and to carry out risk assessments for drug substances commonly administered to stroke patients.

**Methods:**

Drugs frequently administered via feeding tubes were identified through a retrospective analysis of discharge letters from a stroke unit. Physicochemical, pharmacokinetic, and stability properties of these drugs and data on drug-enteral nutrition interactions were systematically searched for in the European Pharmacopoeia, Hagers Handbook of Pharmaceutical Practice, Birchers clinical-pharmacological data compilation, and the Martindale Complete Drug Reference, as well as from databases including DrugBank, DrugDex, PubChem, Google Scholar, and PubMed.

**Results:**

Of the drugs most commonly administered via feeding tubes in the present stroke patient cohort, bisoprolol, candesartan, and ramipril could be considered the least critical due to their overall favourable properties. Acetylsalicylic acid, amlodipine, hydrochlorothiazide, omeprazole and esomeprazole, simvastatin, and torasemide pose risks based on pH or light-dependent instability or proposed food effects. The most critical drugs to be administered via feeding tubes are considered to be furosemide, levodopa, and levothyroxine as they show relevant instabilities under administration conditions and substantial food effects; the latter two even possess a narrow therapeutic index. However, little information is available on drug-tube and drug-formula interactions.

**Conclusion:**

Feeding tube administration of medications turned out to be a highly complex process with several unmet risks. Therefore, investigations that systematically assess these risk factors using clinically relevant model systems are urgently needed.

**Supplementary information:**

The online version contains supplementary material available at 10.1007/s00228-024-03723-4.

## Introduction

Tube feeding is an inevitable requirement once patients are no longer able to take in sufficient amounts of nutrients. Very often, such patients can also no longer take solid dosage forms orally, so these are also administered via the feeding tube. In many cases, this type of administration is not authorised but, nevertheless, practiced due to a lack of alternatives. Several studies have been carried out and recommendations inferred on the administration of medicines via feeding tubes [1–3]. Liquid dosage forms should preferably be used [4]. If such formulations are not available for the drug in question, solid dosage forms should be manipulated in such a way that they can be administered via the tube. This is usually done by crushing and/or suspending. However, strict care must be taken to ensure that the integrity of the dosage form with regard to the intended in vivo release behaviour is not compromised by this manipulation or by administration via the gastric tube, as this would pose a risk to the patient. In the case of modified-release dosage forms, it is important to exclusively apply multiparticulate formulations (dosage forms that break down into smaller units with intact release behaviour when suspended), which must not be further fragmented under any circumstances. To ensure safe and effective administration of the medication, various guidelines emphasize the importance of flushing the tube before, between, and after drug administration [1, 2, 5]. Moreover, flushing the syringe after administration of the medication is also mentioned in several of the publications [2, 3, 5].

Hospitals and care facilities often have internal guidelines for drug administration through feeding tubes, which can differ greatly from each other and from the published recommendations [6]. The deviation from these recommendations might be due to a lack of knowledge of the guidelines and a lack of standardised handling procedures or practical reasons [7–9]. Recommendations in current guidelines, however, do not address the situation after the drug product has been administered from the syringe into the tube, and it is assumed that safe and effective drug therapy is achieved if proper administration (including preparation of the drug product and measuring of the dose) and flushing is considered. However, the whole drug administration process through feeding tubes is more complex. In everyday practice, different nutrient formulas, different feeding regimens (continuous, intermittent, bolus), and different types of feeding tubes (nasogastric or gastrostomy tubes) consisting of different materials (silicone, polyurethane, polyvinyl chloride) with different geometries (length, diameter, connector) are used and combined with different accessories (centric or eccentric syringes, low dose tip, extension systems). It should also be noted that the active ingredients administered via this route can differ significantly in their properties (solubility, pK_a_, logP, etc.) and that variations in the administration of these drugs via feeding tubes can have very different effects on the therapeutic success.

The aim of this work was, therefore, to reflect on the entire process from the selection of the drug to be administered to absorption and to consider what influences the drugs are exposed to at each individual step and what implications this may have for the efficacy and safety of the drug therapy. As many stroke patients require enteral feeding, at least temporarily, and medication is also administered via a feeding tube during this phase, stroke patients represent a very relevant patient group which is why they were selected for this study. Feeding tubes can be placed in the stomach or jejunum. In this study, we focused on gastric feeding tubes as these are preferred over jejunal feeding tubes. Thus, we could collect more data about drugs with evidence of frequent administration as solid dosage forms via feeding tube. Potential risks associated with feeding-tube administration were assessed based on the physicochemical properties of the drugs concerned, and a comprehensive literature search on known risks associated with the way these drugs are administered (e.g. via feeding-tube, with different foods) was conducted.

## Material and methods

### Establishment of a flowchart for establishing risk factors in the process of administering oral medicines via feeding tubes

Based on recommendations available in the literature for the administration of oral drugs via feeding tubes [1–3], a flowchart was created to systematically identify those factors that could compromise safe and effective drug therapy during the administration process. To take into account the complete “journey” of the drug until absorption, the flow chart was extended beyond the end of the administration recommendations (the drug is administered into the tube) until the drug in question reaches its site of absorption (typically the small intestine). In addition to identifying all possible critical points and discussing possible consequences for drug therapy, the flowchart should also be used to identify possible countermeasures that could ensure safe and effective therapy.

### Retrospective data collection of tube-fed patients on a stroke unit

In order to be able to carry out a risk assessment, in November 2016, the IT department at Greifswald University Hospital provided discharge letters from patients on the stroke ward who had undergone a percutaneous endoscopic gastrostomy (PEG) during their stay. All shared data had already been anonymised at this point. From the discharge letters, the following data were extracted: age and sex, diagnoses, home and discharge medication (drug substance, dosage, dosing times) of the patient, and details of the feeding tube used. Discharge letters were excluded from the evaluation if no information on the PEG tube used was available, the feeding tube had been removed during the hospitalisation, the patient was not supplied with an enteral feeding tube, or when the patient had died in the hospital. The remaining discharge letters were included into the evaluation.

For patients whose discharged letters were included into the evaluation, the medication was examined more closely. Orally administered drugs at hospital admission and at discharge were identified. If no dosage form was specified in the discharge letter and both oral and parenteral routes of the drug in question were possible, it was assumed that it was administered orally for the worst-case scenario. However, if the information on the medication in the discharge letter was so imprecise that a clear identification of the drug was not possible, the corresponding drug was excluded from the evaluation.

### Literature research on the drugs most frequently administered via feeding tubes in the stroke-unit patients

The retrospective data evaluation revealed which drugs were most frequently prescribed and thus also most frequently administered via a feeding tube to the patient population studied. A literature search for these drugs was conducted to collate the available information on the drug substances’ chemical, physicochemical, and pharmacokinetic properties, stability data, food effects, and interactions with enteral nutrition.

In the first step, data were collected from the European Pharmacopoeia, Hagers Handbook of Pharmaceutical Practice, Birchers clinical-pharmacological data compilation, and the Martindale Complete Drug Reference, as well as from databases including DrugBank, PubChem, and DrugDex. In the second step, a systematic search for publications was conducted in PubMed. For each drug substance, the following search terms were utilised: drug name (dn) AND interaction AND food, dn AND food-drug-interaction, dn AND pharmacokinetics, dn AND solubility, dn AND pharmacokinetic AND human AND oral, dn AND solubility AND human AND oral, dn AND pka AND solubility, dn AND pharmacokinetics AND pka, dn AND fasted AND fed, dn AND fasted AND food, dn AND feeding tubes, dn AND enteral feed. If the number of hits generated by a certain search term was too high, the corresponding search term was extended by human AND oral. All hits were individually checked for suitability. Additional publications were identified by checking the references of the assessed publications or by using the “similar articles” function in PubMed. The first and second steps were conducted from November 2016 until April 2017. In the third step, in 2022, in addition to the already established data set, information on stability was searched in PubMed and Google Scholar using the search terms: dn AND stability, dn AND stability AND aqueous, dn AND stability AND suspension, dn AND stability AND water, dn AND stability AND syringe, dn AND stability AND photolysis, dn AND stability AND oxidation, dn AND stability AND hydrolysis. Of all hits, only those were considered for further work that related to oral or injectable preparations of the active substances in question (no combination products) and were scientifically sound.

## Results

### Identification of factors influencing the success of drug therapy when administering oral medicines via feeding tubes with the aid of a flowchart of the administration process

**Fig. 1:**
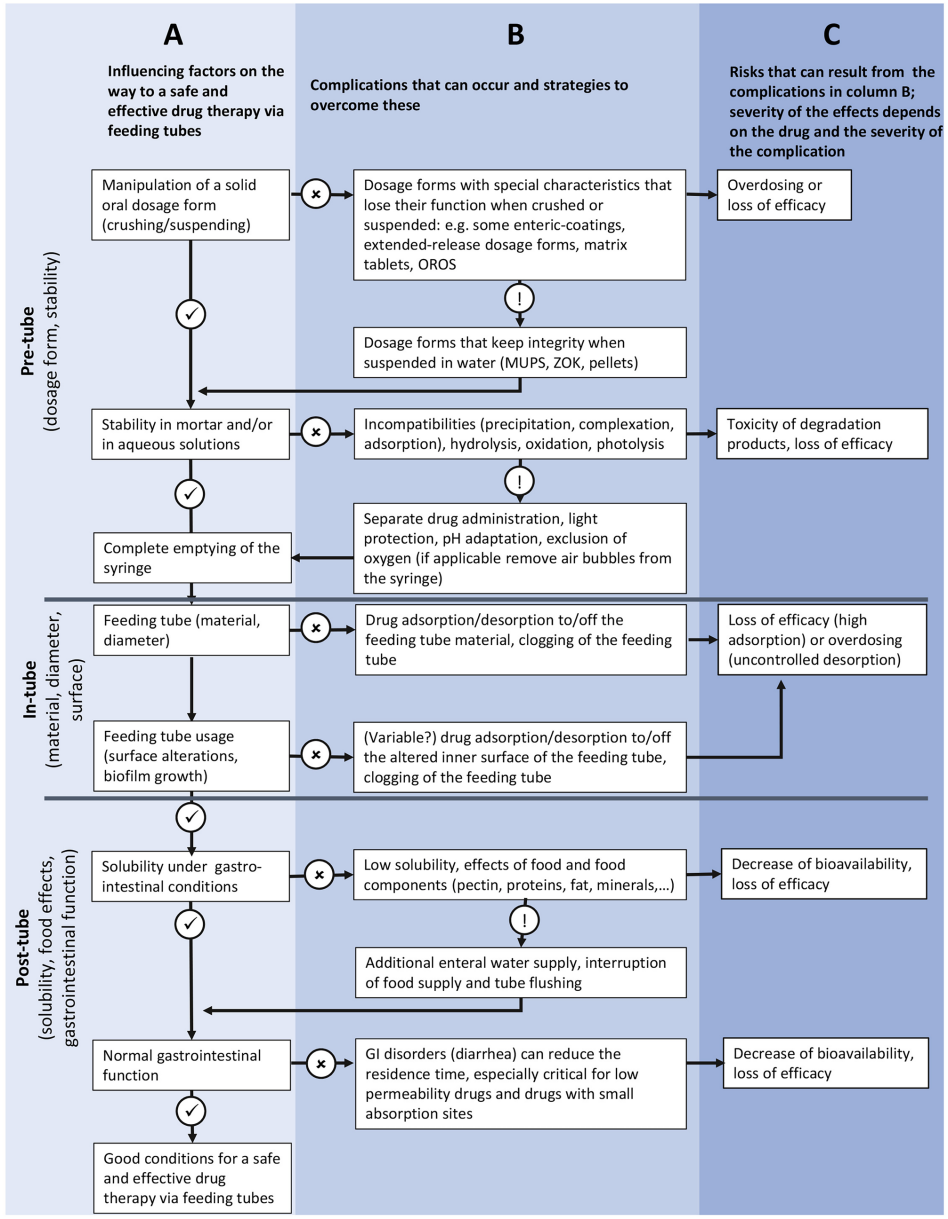
Flow chart of the medication administration process from the drug (products) point of view. OROS, osmotic-controlled release oral delivery system; MUPS, multi-unit pellet system; ZOK, zero-order kinetic; GI, gastrointestinal

Based on information from various literature sources, the administration process varies across institutions. When creating the flowchart depicting drug administration via feeding tubes from the perspectives of the drug substance and the product (Fig. 1), three main phases of the administration process were identified:

The pre-tube phase involves selecting a suitable dosage form and ensuring the stability of the drug (product) in aqueous solutions or as a suspension in a syringe. Monolithic dosage forms must be crushable and/or suspendable for this purpose. A monolithic dosage form is a type of pharmaceutical formulation in which one or multiple active ingredient(s) are uniformly distributed throughout a single, solid matrix. Common examples of monolithic dosage forms include “traditional” tablets or capsules. Some dosage forms with modified release properties are not suitable for administration via a feeding tube, as this would impair their release characteristics, which in turn would be a risk factor for overdosage or loss of efficacy. Accordingly, if modified-release dosage forms are to be administered, it is important to ensure that these are forms in which the controlled release originates from multi-particulate systems, such as the multi-unit pellet system (MUPS). Monolithic dosage forms with this designation can be dispersed in water prior to administration without loss of integrity and while maintaining the release behaviour of the pellet system. Not only can the dissolution/release behaviour be affected, but the preparatory step of supersaturating the formulation of the drug in a syringe can cause stability problems, which can be caused, for example, by sensitivity to light or oxygen and can lead to a drug degradation resulting in a decrease in the drug content even before administration or, in the worst case, to the formation of toxic degradation products. Degradation is a time-dependent process, so immediate administration would be preferred. But in clinical practice, often the drug products are prepared in advance and not at the bedside. Depending on the stability of the suspended drug product, the extent of degradation by light or oxygen can differ. The administration of too low a dose of the drug can also be caused by the drug adsorbing to the syringe material or incomplete emptying of the syringe.

The pre-tube phase is followed by the in-tube phase. As soon as the drug is administered into the feeding tube, interactions with the enteral access devices and/or enteral nutrition residues can occur. These can include tube occlusion because of a too-small diameter or drug adsorption to the feeding tube material. The amount of drug adsorbed to the tube material is (at least temporarily) not available for intestinal absorption. However, since the tube is used again for its original purpose after administration, namely the administration of enteral nutrition, desorption of the drug by rinsing the tube with enteral nutrition formula is conceivable. Furthermore, the inner surface of the feeding tube can change due to food residues and biofilm formation and is therefore subject to constant changes, so that fluctuations in the adsorbed and desorbed quantity are conceivable. Depending on the extent of adsorption/desorption and the therapeutic index of the drug, the risk associated with its enteral administration is more or less pronounced.

The final phase of the administration process begins when the drug leaves the feeding tube. As with the oral administration of solid oral dosage forms, the drug must be dissolved at the site of absorption in order to be absorbed. Low solubility of the drug, changes in solubility due to feeding, and the interaction of drugs with food components can affect the extent and rate of drug absorption. Additional water supply, interruption of the food supply, and tube flushing can be measures to optimise drug efficacy. Patients fed by the enteral route often suffer from diarrhoea that leads to a reduced residence time of the drugs in the gastrointestinal tract. This is of disadvantage for drugs with low permeability and for drugs with small absorption sites. A decrease in absorption and, consequently, a loss of efficacy can be the consequences.

If all these theoretically possible complications could be avoided or minimised, this would provide suitable prerequisites for a safe and effective drug therapy via feeding tubes.

### Drugs administered through feeding tubes in a stroke unit

**Fig. 2:**
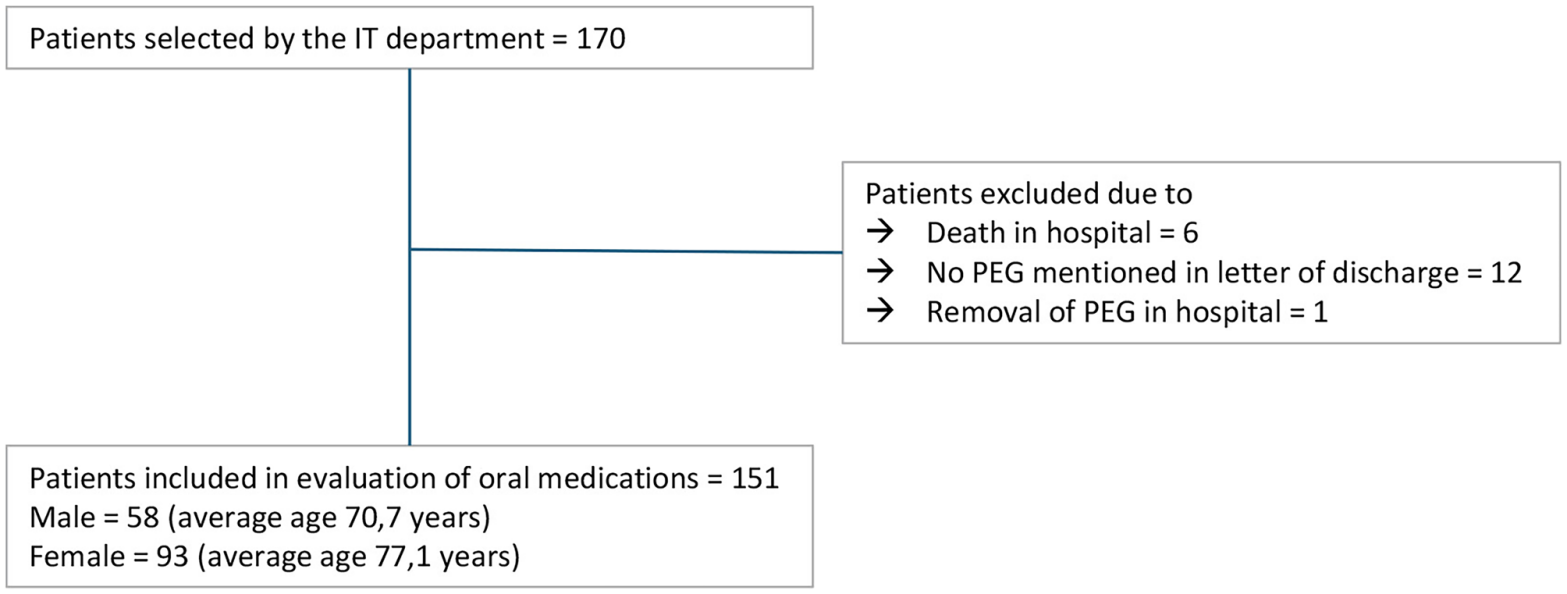
Flow chart showing the results of the inclusion/exclusion process for the selection of patients for data analysis

A flow chart of the selection process for letters of discharge that were evaluated is shown in Fig. 2. From 170 patients originally selected by the IT department, letters of discharge of 151 patients were included in the evaluation of medication.

The most frequently used oral medications in the investigated patient cohort are listed in Table 1, sorted by the absolute number of patients who received these medications. These drugs are normally administered as immediate-release formulations through the feeding tube. Omeprazole and esomeprazole are only available with an enteric coating due to the acid-labile properties of the drug substances. The table also shows the usual dosage as well as the dosage range and dosing schedule in this population.

**Table 1:** The most frequently prescribed oral medications including the most frequent dosage, the dose range, and the dosing schedule of 151 stroke-unit patients with a PEG tube

**Rank**	**Absolute number**		**Drug substance**	**Dosage (range)**	**Dosing schedule**
1	137		Simvastatin	40 mg (10–60 mg)	Once daily; in the evening or before bedtime
2	136		Acetylsalicylic acid	100 mg (25–300 mg)	Once daily; in the morning
3	124		Bisoprolol fumarate	5 mg (1.25–10 mg)	Once or twice daily; in the morning or additionally in the evening
4	84		Amlodipine besylate	5 mg (2.5–10 mg)	Once or twice daily; in the morning or additionally in the evening
5	70		Ramipril	5 mg (2.5–10 mg)	Once or twice daily; in the morning or additionally in the evening
6	59		Torasemide	10 mg (2.5–20 mg)	Once daily; in the morning
7	59		Hydrochlorothiazide	25 mg (12.5–25 mg)	Once daily; in the morning
8	57	44	Esomeprazole	40 mg (20–40 mg)	Once daily; in the morning or before bedtime
		13	Omeprazole	20 mg (20–40 mg)	Once daily; in the morning
9	51		Candesartan cilexitil	16 mg (4–32 mg)	Twice daily; in the morning and in the evening
10	32		Furosemide	40 mg (10–200 mg)	Once or twice daily; in the morning or additionally at noon
[…]					
14	27		Levothyroxine sodium	100 µg (25–150 µg)	Once daily; in the morning
15	22		Levodopa	100 mg (50–200 mg)	individually

### Literature review for the most commonly prescribed drugs in the investigated population

A literature review and risk assessment were conducted for the first 10 drug candidates in Table 1. Furthermore, levothyroxine sodium and levodopa were included in the risk assessment. Since interactions with food and stability problems after oral administration are widely known for these two drugs, they are also likely to occur with enteral administration.

The initial literature search (steps 1 and 2) in the European Pharmacopoeia, Hagers Handbook of Pharmaceutical Practice, Birchers clinical-pharmacological data compilation, Martindale Complete Drug Reference, PubChem, DrugBank, Drug Dex, and PubMed focused on physicochemical and pharmacokinetic properties, interactions with food/enteral nutrition and information about feeding tube administration. The second literature review (step 3) was conducted in PubMed and Google Scholar and focused on the stability of the selected drug substances in the context of administration via feeding tubes. Photolysis, hydrolysis, and oxidation sensitivity were additional interests.

The overall aim of the various steps of the literature search was to obtain an overview of the available information on important aspects of the administration of drugs of interest via feeding tubes. Information on stability aspects was particularly important for a risk assessment of the pre-tube phase. Data from previously published in vitro (determination of recovery) or in vivo studies (measurements of plasma levels or surrogate parameters) on the administration of drugs with the corresponding active substances via nasogastric feeding tubes or PEGs were important for estimating interactions with the tube material and drug loss within the tubes (phase in the tube). Information on interactions with oral or enteral nutrition was important to assess the effect of possible drug-enteral nutrition interactions on the amount of drug available for absorption.

The data acquired on the physicochemical and pharmacokinetic properties of the drugs are summarised in Table 2. The compilation of the relevant literature determined within the framework of the overall literature review can be found in Table 3. The particularities of the individual drugs that should be considered within the framework of a risk assessment as a result of relevant literature data are discussed in the following section.

**Table 2:** Selected physicochemical properties and pharmacokinetic parameters of the most commonly used drugs in the group of stroke-unit patients studied

	**Physicochemical properties**				**Pharmacokinetic parameters**		
	**pK**_**a**_	**logP**	**Solubility in water (15–25 °C)**	**BCS class**	**Plasma protein binding**	**Volume of distribution**	**Oral bioavailability**
**Acetylsalicylic acid**	3.5–3.8 [10–13]	1.18–1.24 [11, 13]	1–10 mg/ml [14]	1 [15], 3 [16]	80–95% [17, 18]	0.15-0.17 1/kg [17, 19]	20–70% [10–12, 17]
**Amlodipine besylate**	8.6–9.3 [11, 20, 21]	3 [11, 13]	1–10 mg/ml [14]	1 [22]	93–98% [11, 13, 17, 19, 20, 23, 24]	20–21 l/kg [11, 13, 17, 20, 23]	52–90% [11, 13, 17–20, 23, 24]
**Bisoprolol fumarate**	9.5 [11, 13]	2.2 [11, 13]	> 1000 mg/ml [14]	1 [25]	26–36% [10, 11, 13, 17–19, 26]	3–3.2 l/kg [10, 17]	80–94% [10, 11, 13, 17–19, 26–28]
**Candesartan cilexetil**	5.3 (candesartan) [29]	6.1 [11, 13]	< 0.1 mg/ml [14]	2 [30]	> 99% [11, 13, 18, 19, 29, 31–33]	0.13 l/kg [11, 13, 19, 29, 31, 33]	15–40% [11, 13, 19, 29, 31–33]
**Esomeprazole**	4.8 [11]	0.6 [11, 13]	1–10 mg/ml (magnesium) [14]	2 [34]	97% [11, 13, 19, 35]	0.2 l/kg [36]	64–90% [18, 35]
**Furosemide**	3.5–3.9 [11, 12, 19, 37–39], 7.5 [11]	2.0 [11, 13]	< 0.1 mg/ml [14]	4 [16]	91–99% [11–13, 17–19, 37, 38]	0.1–0.2 l/kg [11, 13, 17, 19, 38]	47–70% [11–13, 17–19, 37]
**Hydrochlorothiazide**	7.0–9.5 [11, 13, 37, 39]; 9.2–11.3 [11, 37]	−0.07–0.1 [11, 13, 37]	0.1–1 mg/ml [14]	3 [16]	40–68% [11, 13, 17, 19, 37]	0.8–3 l/kg [17, 37]	60–80% [11, 13, 17–19, 37]
**Levodopa**	2.3 [11]; 2.3, 8.7, 9.7, 13.4 [37]	0.1 [11, 13]	1–10 mg/ml [14]	1 [16]	< 10% [17]	0.9–1.6 l/kg [17, 37]	15–33% [17, 37]
**Levothyroxine sodium**	7.4, 9.4 [11]	4 [11, 13]	0.1–1 mg/ml [14]	1 [40], 3 [16]	> 99% [11, 13, 17, 19]	0.1–0.2 l/kg [17]	31–85% [37, 41–45]
**Omeprazole**	4–4.8 [11, 13, 37]; 9.3 [11, 13]	2.2 [11, 13]	0.1–1 mg/ml [14]	2 [46]	95–96% [11, 13, 17, 19, 37]	0.3–0.4 l/kg [11, 13, 17, 19, 37]	30–60% [11, 13, 17, 19, 37]
**Ramipril**	3.7, 5.2 [11]	2.9 [11, 13]	10–33,3 mg/ml [14]	1 [47]	73% [11, 13, 19, 48, 49]	7 l/kg [17] (active metabolite)	28–66% [11, 13, 17–19, 50, 51]
**Simvastatin**	14.9 (acid), − 2.8 (base) [13] (predicted properties)	4.7 [11, 13]	< 0.1 mg/ml [14]	2 [52]	95% [11, 13, 17–19, 53]	-	< 5% [11, 13, 17–19, 49]
**Torasemide**	6.4–7.1 [11, 13, 49]	3.4 [11, 13]	< 0.1 mg/ml [14]	1 [54]	≥ 98% [11, 13, 17, 49, 55, 56]	0.2 l/kg [11, 13, 17, 49, 56]	80–90% [11, 13, 17, 49, 55, 56]

*pKa* acid dissociation constant, *logP* octanol–water partition coefficient, *BCS* Biopharmaceutical Classification System

**Table 3:** Compilation of relevant literature on aspects/parameters relevant to the administration of the corresponding drugs via feeding tubes

	**Stability**				**Feeding tube administration**		**Food impact**	
	**Photolysis**	**Oxidation**	**Hydrolysis**	**Practice-oriented studies**^a^	**Nasogastric**	**PEG**	**Oral food**	**Enteral nutrition**
**Acetylsalicylic acid**	Degradation under UV light in aqueous solution [57] and solid state (degradation rate of 1.7% per second [58])		Sensitive to hydrolysis [12]; hydrolysis to salicylic acid and acetic acid [11] Half-life of 149–325 h in distilled water [59–61] 1–4% of the drug had been hydrolysed after 7 days [62] Increasing pH accelerates hydrolysis [63]	In aqueous suspension tablet lubricants like calcium and magnesium stearate can accelerate hydrolysis of acetylsalicylic acid [64] Acetylsalicylic acid does not adsorb to polyethylene, polystyrene, or nylon syringes [65]			Reduced absorption rate in fed subjects [66–68], but no differences in the bioavailability [67]; delay of absorption observed for solid tablets but not for soluble tablets [19]	
**Amlodipine besylate**	Insensitive to light [20] Sensitive to light [28]; degradation of powdered drug exposed to the light of ID65 standard [69]; half-life of 60 h in powdered tablets when exposed to D65 daylight lamp radiation [70]; raw material showed greater degradation than tablets [71] Degradation under UV irradiation in methanolic solution [72] and in water [73]; degradation in aqueous solution is moderate when irradiated with UV/Vis light for 14 days (forced degradation [74]); half-life in ultrapure water is shorter at higher pH values under solar irradiation [75]	Sensitive to oxidation [28]; degradation under oxidative stress (forced degradation [74, 76])	More degradation in acidic solution than in water [77]; stronger degradation in acidic media than in alkaline media [72]; under forced degradation most instable under alkaline hydrolysis, also instable under acid hydrolysis [74, 76]				Food intake does not affect absorption [11, 13, 17–20]; no significant difference of AUC in healthy subjects [24]; no significant differences in pharmacokinetic parameters in healthy volunteers when dispersed in applesauce [78]; no effect of food on fixed-dose combinations of amlodipine with other antihypertensive drugs [79] or atorvastatin [80] in healthy subjects	
**Bisoprolol fumarate**	Stable in pure water [81], in methanolic solution and solid state under UV light (forced degradation [82] and under a combination of natural daylight and artificial light (forced degradation [83]) Degradation in aqueous-organic solution when exposed to direct sunlight for 72 h (forced degradation [84]) and in solid form under UV light (forced degradation [85])	Stable under oxidative stress (forced degradation [82]) Slight degradation under oxidative stress (forced degradation [84]), formation of degradation products (forced degradation [83, 85])	Stable in water [77], neutral solutions ([86], forced degradation [82, 83]), and alkaline media ( forced degradation [82]) Degradation occurs in acidic environment ([77, 87], forced degradation [82]) and alkaline environment ([86], forced degradation [83–85])				Food does not affect absorption [11, 13, 19, 26, 27]	
**Candesartan cilexetil**	Stable in organic solvent (forced degradation [88] or solid state (forced degradation [89] under light according to ICH guideline for photostability testing or in solid state under sunlight for 14 days (forced degradation [90]) Degradation in solutions with different pH values under light exposure (forced degradation [89])	Stable under oxidative conditions (forced degradation [89]) Slow degradation under oxidative stress (forced degradation [88, 90])	Stable in neutral environment (forced degradation [90]) Hydrolyses into its active form candesartan in neutral environment [91]; more degradation in acidic pH after 1 week than in more neutral pH ranges [91]; fast degradation in acidic media, slower degradation in more neutral media over 14 days at different temperatures [92]; degradation under acid and alkaline conditions (forced degradation [90]); faster degradation under alkaline than under acidic conditions ([93], forced degradation [88]); partly different degradation products under acidic, neutral and alkaline conditions (forced degradation [89])				Food does not affect absorption/bioavailability [11, 13, 29, 31, 32]	
**Esomeprazole**	No degradation under ICH guideline conditions (forced degradation [94])	Minor degradation under oxidative stress (forced degradation [94])	No degradation in acidic environment (forced degradation [94]) Degradation under acidic conditions [95]	Mixture of different PPI was stable for 24 h in 0.9% sodium chloride solution in polypropylene syringes at room temperature with and without light exposure [96]; stable in sachet formulation for up to 60 min after reconstitution [97]; stable in capsule content suspended in tap water diluted with hydrochloric acid to mimic stomach conditions [98]; stable in 0.9% sodium chloride, 5% dextrose and lactated Ringer’s injection in PVC bags over 3 days at room temperature and over 5 days refrigerated (loss less than 7%, [99])	Full recovery of esomeprazole after administration through 8 French nasogastric feeding tubes [94]; good recovery as pellet suspensions administered through enteral feeding tubes with an outer diameter from 6 to 20 French (≥ 96% [97]) Good acid resistance when esomeprazole granules suspended in water were administered through 8 French polyurethane feeding tubes immediately after preparation of the suspension, partly significant drug release in acid media when administration was delayed by 15 min [100] Esomeprazole sachet formulation administered through different tubes on several consecutive days under simulation of enteral nutrition supply showed one block in the smallest feeding tube on the sixth day of the in vitro experiment [101] High delivery rate of esomeprazole pellets delivered through nasogastric feeding tubes via tap water (99% through 14 French tubes, 91% through 8 French tubes [102]) Similar bioavailabilities when esomeprazole is taken orally or administered through nasogastric tubes [103]	Over 99% of esomeprazole pellets could be delivered through 20 French PEG tubes when tap water was used for suspension [102]	Food increases *t*_max_ and decreases the absorption but does not have a significant impact on intragastric acidity [18] Food decreases AUC [19, 35, 104, 105]	
**Furosemide**	No degradation in solid state under direct sunlight [106]; stable in aqueous solutions with pH 8–10 for 24 h when exposed to normal laboratory light/diffuse daylight [107] Sensitive to light [12]; photolysis possible because of its absorption of UV light > 290 nm [11]; discolouration under exposure to light [37]; rapid photolysis in acidic solution [106–108]; degradation in 5% dextrose solution under UV light (254 nm) [109]; fastest degradation rate in solution under long-wave UV light [110]	Little degradation under oxidative stress (forced degradation [111])	Stable in alkaline and neutral solutions ([108, 112], forced degradation [113, 114]) Slow hydrolysis in alkaline environment [37], acidic environment catalyses degradation [108, 112–114]; half-life in acidic solution: 17.2 h [107]; most degradation under alkaline stress conditions (forced degradation [111])	Stable when dissolved in 0.9% sodium chloride or 5% dextrose solution in polypropylene syringes with and without light exposure for at least 24–72 h [111, 115, 116]; stable in minibags and polypropylene syringes for 84 days when protected from light and for additional 7 days when exposed to fluorescent light [117]			Bioavailability is similar regardless of the dosage form when administered as a tablet or aqueous solution in a fasted and fed state in healthy volunteers [118] Food slightly decreased bioavailability (not significant) [119]; a decrease of bioavailability in healthy subjects when given with breakfast [120, 121]; or in patients after breakfast [122]; food may have an influence on the urinary output in patients with oedema → more studies in patients are needed [123] Bioavailability of furosemide is influenced by food and other factors [18] Decrease of bioavailability when administered with food [17, 19]	
**Hydrochlorothiazide**	Stable under sunlight for 14 days (forced degradation [90]) and under photolytic stress conditions according to the ICH guideline (solid drug) (forced degradation [124, 125]) Degradation under UV light [126]	Stable under oxidative stress (forced degradation [90]) Degradation under oxidative stress (forced degradation [124, 125])	In aqueous solution cleavage of formaldehyde, most stable at pH 7.2 [37]; faster degradation with increasing pH [37, 126] Degradation under neutral and acid conditions but mostly under alkaline conditions (forced degradation, [90]); degradation in acidic and alkaline (forced degradation [124, 125]) and neutral solutions (forced degradation [124])				Hydrochlorothiazide does not show any food effect [39] Decrease of rate and extent of hydrochlorothiazide absorption together with food [11, 19]; multivalent cations reduce absorption [13]; in healthy volunteers, food intake reduced plasma drug levels significantly [127] No significant difference in the area under the curve of fasted and fed volunteers, but significantly more urinary recovery in fed patients → food enhances absorption of hydrochlorothiazide [128] Concomitant food intake increases bioavailability [17]	
**Levodopa**	Stable as levodopa/carbidopa solutions under ambient light [129]; stable in (acidic phosphate buffered) solution under UV-A radiation (forced degradation [130, 131]) Instable under UV-C radiation (forced degradation [130])	Oxidation by atmospheric oxygen [11, 18, 37]; (mild) degradation under oxidative stress conditions (forced degradation [130, 131])	Stable in acidic solution (forced degradation [130, 131]) Degradation in alkaline media (forced degradation [130, 131])	Stable in SGFsp for 7 days [132] Degraded to a small extent as levodopa/carbidopa dissolved in tap water [132]			Reduced absorption with concomitant food intake [17, 19, 28] Reduced absorption with concomitant food/protein intake due to competition with amino acids for transporter capacities [19, 37]; competition with amino acids for transport into the brain [19] Correlation between protein intake and severe motor fluctuations could be shown in patients with Parkinson’s disease [133] No impairment of rate or extent of levodopa absorption by food in healthy volunteers and patients → beneficial effects of low protein diet in patients with Parkinson’s disease seem to result from interactions with amino acids at the blood–brain barrier and not from competitive intestinal absorption [134, 135]	Case reports where enteral-fed patients received levodopa and high protein nutritional formula resulting in severe rigidity and neuroleptic malignant like syndrome; reduction of protein content lead to improvement of the symptoms [136, 137]
**Levothyroxine sodium**	Very slow degradation in aqueous solution under solar irradiation [138] Degradation under sunlight and artificial room light in methanolic-aqueous solution [139] Instable in aqueous solution under UV radiation [138, 140]; degradation within approximately 16 days (forced degradation [141])	Degradation of levothyroxine under oxidative stress (forced degradation [141])	No hydrolysis is expected in the pH range of 5–9 [11] More stable in acidic than in neutral or alkaline environment [139] Slower degradation in alkaline than in neutral or acidic media [142] Most instable under alkaline conditions compared to acidic stress conditions (forced degradation [141])	Stable over 24 h in different infusion containers when powder is reconstituted with 0.9% sodium chloride solution and further diluted with 5% dextrose solution, ad- and desorption to/from PVC [141]; stable over 24 h in 0.9% sodium chloride solution and stored under fluorescent lightning and protected from light at room temperature [143]; in 0.9% sodium chloride solution in polypropylene syringes at 5 °C, no adsorption onto the syringe; drug was stable for 7 days [144] Not stable for 24 h when dissolved in 0.9% sodium chloride solution and stored in PVC mini bags [145]; stability of suspended tablets is limited at lower pH [146] Adsorbed by polypropylene tubing when powder was reconstituted with 0.9% sodium chloride, further diluted with 5% dextrose solution, and passed through 40 cm of polypropylene tubing under fluorescent light exposure [147]	No differences in TSH, fT4, and fT3 comparing levothyroxine tablet (pause of enteral nutrition supply) and liquid formulation (continuous enteral feeding) in patients with nasogastric feeding tube [148]	Extent of adsorption to PEG feeding tube is probably clinically insignificant [149]	Reduced absorption when administered with food [18, 37] Reduction of levothyroxine absorption by multivalent cations like calcium, iron, and magnesium [11, 18, 42, 150, 151], soy protein-containing food supplements or formula [13, 19, 152, 153], and dietary fiber-containing supplementation [11, 13, 19, 154] Delay of absorption with only little effect on bioavailability by grapefruit juice [41]	Soybean-based formulas can impair intestinal reabsorption and lead to an increase in elimination [19] Patients under levothyroxine replacement therapy and continuous enteral nutrition can develop hypothyroidism and should therefore be monitored closely [155]
**Omeprazole**	Stable under UV and fluorescent light exposure for 1 week (forced degradation [156]) Degradation under the exposure to sunlight [157]	Oxidation occurs (forced degradation [156, 158])	Instable under acid conditions [95, 157, 159]; stability improves with increasing pH [159–161]; degradation in acid, neutral, and alkaline environment (forced degradation [156]); extensive degradation under acid conditions (forced degradation [158])	Stable in a mixture of different PPI in 0.9% sodium chloride solution in a polypropylene syringe for 24 h [96]; stable in 8.4% sodium bicarbonate solution and stored in 5 ml amber-coloured plastic oral syringes at room temperature for 14 days [162] and in the refrigerator for 45 days [162, 163]; dissolved capsule content in 8.4% sodium bicarbonate was stable in amber-coloured glass bottles at 5 °C storing conditions for 28 days [164]; stable in tap water stored in polypropylene tubes in darkness for up to 28 days [165]; suspended in SGF for 2 h [163]; compounded suspension by grinding was stable for 1 week, compounded suspension by shaking was stable for 4 weeks [166] Degradation of capsule content dissolved in 8.4% sodium bicarbonate solution and stored at room temperature for 30 days [167]; faster degradation in 5% dextrose solution compared to 0.9% sodium chloride solution in infusion bags and stored under conditions like in a normal ward [168] Differences in stability comparing extemporaneously compounded omeprazole suspension from different commercially available solid omeprazole formulations [169]	No significant reduction in recovery after giving compounded suspension through different nasogastric feeding tubes [158, 163]		No influence of food on the bioavailability (AUC) under repeated dose conditions [170] Reduction of the absorption rate but no effect on the extent [19] Single dose of omeprazole enteric-coated pellets showed delayed absorption and lower bioavailability when given with food [171] Reduction of *c*_max_ and AUC when administering omeprazole 1 h after a meal [19]	
**Ramipril**	Stable under UV/Vis radiation (forced degradation [172, 173])	Stable to oxidative degradation ([174], forced degradation [173]) Degradation under oxidative stress (forced degradation [172])	Increasing degradation with increasing pH [174]; more degradation in alkaline media (forced degradation [172, 173])	Stable for 24 h when capsule content is suspended in water at room temperature, for 48 h at 3 °C [175]			Food influences the rate of absorption but not the extent of absorption [11, 13, 19]	
**Simvastatin**	Photostable for 3 h [176] Decrease to 89% under UV light (254 nm) in 60 min, under natural sunlight conversion to hydroxy acid form and degradation [177]	No oxidation (oxygen bubbling, forced degradation [178]) Sensitive to oxidation (H_2_O_2_, forced degradation [179])	Conversion to hydroxy acid form lowest in acidified samples [177]; good stability at pH 5 [180] Hydrolysis under acid, basic, and neutral conditions [179]; fastest degradation rate at pH 2 [180]; increasing instability with increasing pH (forced degradation [178])				No food effect [19, 181]	
**Torasemide**	Photostable (forced degradation [182, 183])	(Slightly) instable (forced degradation [182, 183])	Stable in neutral and alkaline environment (forced degradation [182, 183]) Degradation under acidic conditions (forced degradation [182, 183])	Stable for 72 h in 5% dextrose infusions stored under normal fluorescent light at room temperature [184]			No food effect [17, 185, 186] Decrease in absorption [56, 123] Decreased absorption rate but not bioavailability [19]	

*AUC* area under the curve, *c*_max_ maximum plasma concentration, *D65* daylight, *fT4* free thyroxine, *fT3* free triiodthyronine, *ICH* International Council for Harmonisation of Technical Requirements for Pharmaceuticals for Human Use, *ID65* indoor light, *PEG* percutaneous endoscopic gastrostomy, *PPI* proton pump inhibitor, *PVC* polyvinylchloride, *SGF(sp)* simulated gastric fluid (sine pepsin), *t*_*max*_ time until maximum plasma concentration is reached, *TSH* thyroid-stimulating hormone, *UV* ultraviolet, *Vis* light in the visible spectrum

^a^Practice-oriented refers to stability studies of suspensions of solid oral dosage forms or of injection solutions or to studies on the adsorption of the drugs in question to syringe materials

## Discussion

### Risk assessment

The likelihood of safe and effective administration of drugs via feeding tubes and the risk that this route of drug administration will be the cause of absorption variability leading to ineffective systemic drug concentrations or unintended or toxic side effects depend on several drug-related factors. A higher risk of variability is typically associated with drugs with a narrow therapeutic index. With such drugs, even small variations in the amount of drug taken can have large effects. In addition to the variability caused by the administration process, the solubility of the drug in the gastrointestinal tract is another aspect to consider. Dissolution of the drug in the conditions of the upper gastrointestinal tract is a prerequisite for intestinal absorption of the drug. If the drug has poor or limited solubility under these conditions, the proportion of the dose absorbed may be significantly reduced [187]. Soluble maximum doses of the drug substance in 250 ml over the pH range of 1–6.8 at 37 °C [188] means high solubility in the BCS context. If a drug is categorised in different BCS classes, it is likely that is BCS class I or III and BCS class II or IV, respectively. This indicates varied assessment not of the solubility but of the permeability of the drug substance. Different methods are used to assess the permeability. The assessment should be based on pharmacokinetic data in humans; animal or cell culture data should only be additional [188].

In contrast, according to the European Pharmacopoeia, solubility is determined in water at 15–25 °C [14]. This is why slightly to very slightly soluble drugs like acetylsalicylic acid or hydrochlorothiazide can still be categorised into BCS classes with high solubility. The water solubility of the drugs in question can be used as a further indicator since these or the corresponding drugs are dispersed in water before administration. Good water solubility can prevent solid, unadministered drug residues in syringes and feeding tubes, thus reducing the risk of underdosing.

By categorising the drugs examined in this study according to their therapeutic index and their solubility under gastrointestinal conditions and taking the results of the literature search into account, it should be possible to assess whether their administration via feeding tubes poses a lower or higher risk of deviations in the amount of drug available for absorption and thus ultimately also for the intended efficacy.

### Group 1: drugs with a wide therapeutic index and a high solubility according to the BCS (Class I/III)

Bisoprolol is very soluble in water [14], and stability and food interaction data can be interpreted as unproblematic for feeding tube administration. Nevertheless, the European Pharmacopoeia recommends protection from light and storage in an airtight container for the pure drug substance [14]. No studies were found for feeding tube administration.

According to the European Pharmacopoeia, ramipril is sparingly soluble in water, and the drug substance should be protected from light [14]. In general, stability and food interaction studies indicate an unproblematic feeding tube administration. Further attention should be paid to its degradation in alkaline solutions. The pH of the drug product suspension should be measured to ensure sufficient stability conditions. One practice-oriented study investigated the stability of suspended ramipril capsule content and could show sufficient stability. No studies were found for feeding tube administration.

Acetylsalicylic acid is slightly soluble in water [14]. The European Pharmacopoeia recommends storage in an airtight container [14] since acetylsalicylic acid is sensitive to hydrolysis even in moist air. However, literature data show sufficient stability of the drug substance in suspension in distilled water. One should be aware that tablet lubricants can accelerate the degradation of the drug substance. The adsorption of acetylsalicylic acid to different plastics is unproblematic, but further studies for administration through feeding tubes are missing. Food does not affect bioavailability.

Amlodipine is slightly soluble in water and the European Pharmacopoeia recommends storage protected from light and in an airtight container [14]. Stability data indicate possible problems (photostability, sensitivity to oxidation, and hydrolysis), but data are partly contradictory, or the relevance for feeding tube administration is difficult to assess due to different test conditions (e.g. exposure to sunlight or UV light). No practice-oriented studies and studies for feeding tube administration were found. Food does not affect bioavailability.

Hydrochlorothiazide is very slightly soluble in water [14]. The recommendation to protect hydrochlorothiazide from light was found in the literature [37]. However, study results indicate a sufficient photostability of hydrochlorothiazide for feeding tube administration. For oxidation and hydrolysis, partly contradictory results were found, but there is a tendency towards lower stability of hydrochlorothiazide in alkaline solutions. No practice-oriented studies and studies for feeding tube administration were found. Contradictory literature was found for drug-food interactions. These observations might relate to the pH-dependent stability of hydrochlorothiazide, a question which deserves further investigation.

Torasemide is practically insoluble in water and, according to the European Pharmacopoeia, should be protected from light [14]. Stability data are only generated in forced degradation studies so the relevance for feeding tube administration is not clear. One study investigated the stability of torasemide in 5% dextrose solution under normal fluorescent light and found it to be stable under these conditions. No practice-oriented studies and studies for feeding tube administration were found. Contradictory literature was found for drug-food interactions.

### Group 2: drugs with a wide therapeutic index and a low solubility according to the BCS (Class II/IV)

Esomeprazole is slightly soluble as magnesium salt [14]. The European Pharmacopoeia recommends storage protected from light and in an airtight container [14]. The stability studies found show no problems in terms of sensitivity to light and oxidation. But, as it is widely known, proton pump inhibitors are acid-labile drug substances. Therefore, they have to be enteric-coated. Many practice-oriented stability studies show sufficient stability of feeding tube-suitable pellet/sachet formulations. Additionally, many studies about feeding tube administration were found in the literature, indicating good handling and recovery of the drug product and drug substance, respectively. One study even investigated the bioavailability of esomeprazole administered via a feeding tube compared to oral intake. No difference in bioavailability could be observed in this study. Data about drug-food interactions are contradictory, saying that there is no food effect or a decreased AUC (area under the curve), whereby the reduced AUC, maybe, does not have an impact on intragastric acidity.

Omeprazole is very slightly soluble and should be protected from light and stored in an airtight container in the refrigerator [14]. Stability data indicate that there could be a problem when omeprazole is exposed to light or oxidising agents. The drug substance is definitely instable under acidic conditions and, therefore, has to be enteric-coated. Many practice-oriented stability studies were conducted in different solutions (0.9% sodium chloride, 5% dextrose, 8.4% bicarbonate). They have in common that omeprazole degrades faster under light exposure and at room temperature. However, stability is still good enough for extemporaneously prepared suspension for feeding tube administration. Studies about feeding tube administration show good recovery of omeprazole. It is recommended to administer omeprazole 1 h before a meal [104], but data about drug-food interactions are contradictory.

According to the European Pharmacopoeia, simvastatin is practically insoluble in water and should be protected from light and stored under nitrogen or with antioxidants in an airtight container [14]. Stability studies showed no explicit results regarding stability. No practice-oriented studies and studies for feeding tube administration were found. Food does not affect bioavailability.

Candesartan cilexetil is practically insoluble in water [14]. It is quite stable against photodegradation and oxidation but shows pH-dependent hydrolysis. No practice-oriented studies and studies for feeding tube administration were found. Food does not affect bioavailability.

Furosemide is practically insoluble in water and should be protected from light [14]. Stability studies found in the literature also indicate a sensitivity to light. Furosemide seems to be instable in an acidic environment, whereby there are contradictory results. However, practice-oriented studies indicate sufficient stability for feeding tube administration. Studies about feeding tube administration could not be found. Food seems to have a negative effect on bioavailability.

### Group 3: narrow therapeutic index drugs

According to the European Pharmacopoeia, levodopa is slightly soluble in water and should be protected from light [14]. However, the found literature indicates sufficient photostability. Oxidation was observed but only to a small extent. Levodopa was most stable under acid conditions. One practice-oriented study was conducted to assess the stability of levodopa in tap water. The study showed sufficient stability of levodopa in tap water. Studies about feeding tube administration could not be found. Food with high protein content can influence levodopa therapy.

Levothyroxine sodium is very slightly soluble and should be stored protected from light in an airtight container in the refrigerator [14]. The results of the literature search indicate a sensitivity to light which could be problematic. However, practice-oriented studies resulted in sufficient stability for feeding tube administration. On the one hand, the studies about feeding tube administration do not show a significant or clinically relevant loss of levothyroxine. On the other hand, an increase of the necessary levothyroxine dose to maintain a euthyreot metabolic state was observed when levothyroxine was administered via feeding tubes. Many interactions like complexation of multivalent cations and adsorption to soy bean are known for levothyroxine.

### Conclusion

For some drug substances, much literature was found in the context of feeding tube administration. Omeprazole and esomeprazole have one of the most comprehensive literature on the selected drug substances. This includes many practice-oriented stability studies as well as feeding tube administration studies. This information is needed for a concluding risk assessment. This work shows that there is a lack of these studies for most of the investigated drug substances. In addition, the used drug product can have a great influence on stability and/or feeding tube administration which is why we cannot give any recommendations.

#### Current state and future needs

Typically, for each registered drug substance, there is already some data available on the stability of the pure substance from simple stability studies or forced degradation studies. These provide a first indication of the degradation pathways of the drug but allow only limited conclusions to be drawn about stability in the context of feeding tube administration. In addition to the stability data generated for new active substances/drugs as part of the marketing authorisation process, stability tests for injectable preparations in the appropriate syringes are available for some drugs. The results of such tests already provide a good indication of the stability of the drug when administered via a feeding tube, but the underlying test design differs in the time periods investigated and, in the case of finished drug products, often in the excipients contained in the finished drug product. On the other hand, as solid dosage forms consisting of drug substance and excipients are usually manipulated for administration via the feeding tube, the influence of all excipients contained in the finished drug product would have to be taken into account. Targeted in-use stability studies on the individual manipulated drug products for feeding tube administration would therefore be desirable.

The enteral feeding market offers a wide range of products that may differ in terms of the materials and geometries used. Results of studies with one type of tube and syringe may not be applicable to all other tubes and syringes. The Food and Drug Administration (FDA) Draft Guidance “Oral Drug Products Administered Via Enteral Feeding Tube: In Vitro Testing and Labelling Recommendations” [189] states that the different geometries of feeding tubes should be considered and tested accordingly. It also recommends that at least the smallest diameter for which a feeding tube application is to be approved should be tested. This is consistent with the observations of Shah et al*.*, who investigated how many of the esomeprazole-containing pellets passed through the tube. In this study, more pellets were retained in the smaller tubes [102]. However, in a systematic study by Karkossa et al*.* in which different pellet formulations were administered in vitro through different feeding tubes using different syringes and extension systems, it was shown that the diameter of the feeding tube or pellet size does not allow a clear prediction of the quantitative performance of the administration [190]. In another in vitro study, Karkossa et al*.* determined the recovery of ibuprofen after the administration of various ibuprofen suspensions after the administration via different types of feeding tubes [191]. The results of these experiments also indicated that the diameter of the feeding tube is a poor indicator of whether a given dose of a drug product can be successfully administered. These and a number of other studies of a similar nature [94, 100, 102] clearly show, as already mentioned above, that the success of drug administration via a feeding tube is determined by a complex interplay of the properties of the administered drug and the administered drug product, the geometry and material of the syringe and feeding tube, the volume of the administered suspension/solution, the flushing volume, as well as all preparatory measures and many other details.

As became clear in the present study, in daily practice, many pharmaceutical forms available on the market are administered in manipulated form via enteral feeding tubes without knowing whether this allows safe administration of the intended drug dose. At this point, it is also important to remember that the primary purpose of a feeding tube is to ensure the patient’s nutrition. Accordingly, enteral formulas are fed to the patients concerned via the feeding tube for many hours every day. Although feeding should normally be stopped and the feeding tube rinsed with water prior to drug administration, food residues may accumulate on the inner surface of the tube, altering the tube surface and leading to interactions with the administered drug. The impact of such conditions on the administration process or on dose recovery should also be investigated in in vitro studies where possible. It should further be noted that the nature and extent of such interactions may depend on the type and composition of the enteral formula, as has been demonstrated with levothyroxine [152, 153, 192]. Given the complexity of the issue, it quickly becomes clear that an in vitro simulation of administration cannot fully reflect in vivo conditions. Especially when it comes to estimating the influence of food residues on the successful application of a drug dose, the simulation would have to be taken even further, because in vivo different bacteria also enter the feeding tube, which in turn form biofilms there due to the good nutrient supply. Formation of such biofilms can create a completely different surface structure (from abiotic to biotic) in the lumen of the tube [193]. It would be interesting to also map the impact of biofilms on the success of the administration process of the respective drug in in vitro experiments, but a realistic simulation would probably only be possible in ex vivo experiments with feeding tubes removed from patients after the end of enteral nutrition.

Finally, it is important to check whether the modified drug still exhibits the originally intended release behaviour in the gastrointestinal tract. This is particularly relevant for pellets with modified drug release and has already been investigated in studies with esomeprazole and omeprazole pellets [100, 163] but should also be of interest for other drugs.

#### Limitations

The first and second literature searches on physicochemical and pharmacokinetic properties were conducted from November 2016 to April 2017. Due to limited resources, the additional (third) literature search on stability and practice-oriented information was conducted in 2022. Since it is unlikely that physicochemical and pharmacokinetic properties change, we do not think a repetition of the first and second literature searches was necessary.

## Conclusion

In this work, a flow chart of the administration process of oral drugs via enteral feeding tubes was created, which takes into account all essential steps from the selection of the drug product to the absorption of the administered drug in the gastrointestinal tract. This flow chart can be used to identify critical points in the administration process where drug loss can occur, thereby compromising a safe and effective drug therapy. The new flow chart was used for the first time in this study to carry out a risk assessment with regard to the administration of drugs via feeding tubes using the data available in the literature for the drugs that are proven to be most commonly administered in a specific stroke unit. It was found that the most commonly administered drug products comprise several drug substances that are considered highly problematic for this route of administration, as they have a narrow therapeutic index, are unstable in aqueous solutions or in the presence of light, or are subject to food effects. When the critical points identified in the flow chart and the drug properties determined in the literature are compared with the information available for the administration of the drugs in question via enteral feeding tubes, it is clear that there is a lack of reliable data that can demonstrate safe administration via this route. This highlights the need for systematic studies to assess the risk of this route of administration in terms of safe and effective drug therapy. The established flow chart can provide valuable guidance for the planning of such studies and thereby make a valuable contribution to de-risking the administration of drugs via feeding tubes.

## Supplementary information

Below is the link to the electronic supplementary material.

Supplementary file1 (DOCX 23 KB)

Supplementary file2 (XLSX 116 KB)

## Data Availability

No datasets were generated or analysed during the current study.

## References

[1] Bankhead R, Boullata J, Brantley S et al (2009) Enteral nutrition practice recommendations. JPEN J Parenter Enteral Nutr 33:122–167. 10.1177/0148607108330314 19171692 10.1177/0148607108330314

[2] Boullata JI (2009) Drug administration through an enteral feeding tube. Am J Nurs 109:34-42; quiz 43. 10.1097/01.NAJ.0000361488.45094.28 10.1097/01.NAJ.0000361488.45094.28 21753476

[3] Boullata JI (2021) Enteral Medication for the Tube-Fed Patient: Making This Route Safe and Effective. Nutr Clin Pract 36:111–132. 10.1002/ncp.10615 33373487 10.1002/ncp.10615

[4] Williams NT (2008) Medication administration through enteral feeding tubes. Am J Health Syst Pharm 65:2347–2357. 10.2146/ajhp080155 19052281 10.2146/ajhp080155

[5] Bischoff SC, Austin P, Boeykens K et al (2022) ESPEN practical guideline: Home enteral nutrition. Clin Nutr 41:468–488. 10.1016/j.clnu.2021.10.018 35007816 10.1016/j.clnu.2021.10.018

[6] Joos E, Mehuys E, van Bocxlaer J et al (2015) Drug administration via enteral feeding tubes in residential care facilities for individuals with intellectual disability: an observational study. J Intellect Disabil Res 59:215–225. 10.1111/jir.12129 24762229 10.1111/jir.12129

[7] Bandy KS, Albrecht S, Parag B et al (2019) Practices Involved in the Enteral Delivery of Drugs. Curr Nutr Rep 8:356–362. 10.1007/s13668-019-00290-4 31606851 10.1007/s13668-019-00290-4

[8] Hossaini Alhashemi S, Ghorbani R, Vazin A (2019) Improving knowledge, attitudes, and practice of nurses in medication administration through enteral feeding tubes by clinical pharmacists: a case-control study. Adv Med Educ Pract 10:493–500. 10.2147/AMEP.S203680 31372085 10.2147/AMEP.S203680 PMC6628606

[9] Tillott H, Barrett D, Ruan J et al (2020) Survey of nurses' knowledge and practice regarding medication administration using enteral tubes. J Clin Nurs 29:4614–4622. 10.1111/jocn.15498 32954598 10.1111/jocn.15498

[10] Dannhardt G (1993) Hagers Handbuch der Pharmazeutischen Praxis: Stoffe A-D, 5th edn. Springer, Berlin / Heidelberg, Berlin, Heidelberg

[11] National Center for Biotechnology Information PubChem^®^ PubChem^®^. https://pubchem.ncbi.nlm.nih.gov/. Accessed 04 Dec 2019

[12] Gröning R (1987) Generika: Arzneistoffprofile und Bioverfügbarkeitsdaten von Fertigarzneimitteln. Dt. Apotheker-Verl, Stuttgart

[13] DrugBank. https://go.drugbank.com/. Accessed 28 Dec 2020

[14] Pharmacopoeia European (2022) European Pharmacopoeia, 11th Ed., English: 11.0 - 11.2: Subscription to Main volume + Supplement 1 + Supplement 2, 11. Auflage. Deutscher Apotheker Verlag, Stuttgart

[15] Dressman JB, Nair A, Abrahamsson B et al (2012) Biowaiver monograph for immediate-release solid oral dosage forms: acetylsalicylic acid. J Pharm Sci 101:2653–2667. 10.1002/jps.23212 22674043 10.1002/jps.23212

[16] Lindenberg M, Kopp S, Dressman JB (2004) Classification of orally administered drugs on the World Health Organization Model list of Essential Medicines according to the biopharmaceutics classification system. Eur J Pharm Biopharm 58:265–278. 10.1016/j.ejpb.2004.03.001 15296954 10.1016/j.ejpb.2004.03.001

[17] Bircher J, Sommer W (1999) Klinisch-pharmakologische Datensammlung, 2, völlig neu, bearb. Wiss. Verl.-Ges, Stuttgart

[18] Reynolds JEF (ed) (1996) Martindale - The extra pharmacopoeia; [evaluated information on the world's drugs and medicines], 31st edn. Royal Pharmaceutical Soc, London

[19] Truven Health Analytics Inc. (2016) DrugDex. https://www.micromedexsolutions.com. Accessed 18 Nov 2016

[20] Handrock R, Herzig S (1998) Amlodipin (Amlodipine). Dtsch Med Wochenschr 123:19–20. 10.1055/s-0029-1233207 9465851 10.1055/s-0029-1233207

[21] van Zwieten PA (1994) Amlodipine: an overview of its pharmacodynamic and pharmacokinetic properties. Clin Cardiol 17:III3-6 9156957

[22] Williams HD, Ford L, Lim S et al (2018) Transformation of Biopharmaceutical Classification System Class I and III Drugs Into Ionic Liquids and Lipophilic Salts for Enhanced Developability Using Lipid Formulations. J Pharm Sci 107:203–216. 10.1016/j.xphs.2017.05.019 28549907 10.1016/j.xphs.2017.05.019

[23] Meredith PA, Elliott HL (1992) Clinical pharmacokinetics of amlodipine. Clin Pharmacokinet 22:22–31. 10.2165/00003088-199222010-00003 1532771 10.2165/00003088-199222010-00003

[24] Lv C, Wei C, Wang X et al (2014) The influence of food on the pharmacokinetics of amlodipine and losartan after single-dose of its compound tablets in healthy chinese subjects. Drug Res (Stuttg) 64:229–235. 10.1055/s-0033-1357143 24132705 10.1055/s-0033-1357143

[25] Macwan JS, Fraczkiewicz G, Bertolino M et al (2021) Application of physiologically based biopharmaceutics modeling to understand the impact of dissolution differences on in vivo performance of immediate release products: The case of bisoprolol. CPT Pharmacometrics Syst Pharmacol 10:622–632. 10.1002/psp4.12634 34080804 10.1002/psp4.12634 PMC8213417

[26] Prichard BN (1987) Bisoprolol: a new beta-adrenoceptor blocking drug. Eur Heart J 8 Suppl M:121–129. 10.1093/eurheartj/8.suppl_m.121 10.1093/eurheartj/8.suppl_m.121 2897295

[27] Leopold G (1986) Balanced pharmacokinetics and metabolism of bisoprolol. J Cardiovasc Pharmacol 8(Suppl 11):S16-20. 10.1097/00005344-198511001-00003 2439789 10.1097/00005344-198511001-00003

[28] A. Pfaff Pharmatrix. www.pharmatrix.de. Accessed 24 Jan 2017

[29] Gleiter CH, Jägle C, Gresser U et al (2004) Candesartan. Cardiovasc Drug Rev 22:263–284. 10.1111/j.1527-3466.2004.tb00146.x 15592574 10.1111/j.1527-3466.2004.tb00146.x

[30] Figueroa-Campos A, Sánchez-Dengra B, Merino V et al. (2020) Candesartan cilexetil in vitro-in vivo correlation: predictive dissolution as a development tool. Pharmaceutics 12. 10.3390/pharmaceutics12070633 10.3390/pharmaceutics12070633 PMC7408357 32640620

[31] Gleiter CH, Mörike KE (2002) Clinical pharmacokinetics of candesartan. Clin Pharmacokinet 41:7–17. 10.2165/00003088-200241010-00002 11825094 10.2165/00003088-200241010-00002

[32] Israili ZH (2000) Clinical pharmacokinetics of angiotensin II (AT1) receptor blockers in hypertension. J Hum Hypertens 14(Suppl 1):S73-86. 10.1038/sj.jhh.1000991 10854085 10.1038/sj.jhh.1000991

[33] Jeon J-Y, Im Y-j, Kim Y et al (2013) Pharmacokinetic properties and bioequivalence of candesartan cilexetil in Korean healthy volunteers. Drug Dev Ind Pharm 39:1296–1299. 10.3109/03639045.2012.725732 23030309 10.3109/03639045.2012.725732

[34] Pyo Y-C, Nguyen TN, Lee Y-S et al (2023) Design of esomeprazole solid dispersion for improved dissolution and bioavailability using the supercritical anti-solvent technique. J Drug Deliv Sci Technol 88:104889. 10.1016/j.jddst.2023.104889

[35] Vachhani R, Olds G, Velanovich V (2009) Esomeprazole: a proton pump inhibitor. Expert Rev Gastroenterol Hepatol 3:15–27. 10.1586/17474124.3.1.15 19210109 10.1586/17474124.3.1.15

[36] Heumann Pharma GmbH & Co. Generica KG (2023) Fachinformation esomeprazol heumann 20 mg/40 mg magensaftresistente tabletten. https://www.heumann.de/fileadmin/user_upload/produkte/infos/Fachinformation-Esomeprazol-Heumann-magensaftresistente-Tabletten.pdf. Accessed 03 Apr 2023

[37] Hager H (1993) Hagers Handbuch der Pharmazeutischen Praxis: Band 8: Stoffe E-O, 5th ed. Springer Berlin / Heidelberg, Berlin, Heidelberg

[38] Cvijić S, Parojčić J, Langguth P (2014) Viscosity-mediated negative food effect on oral absorption of poorly-permeable drugs with an absorption window in the proximal intestine: In vitro experimental simulation and computational verification. Eur J Pharm Sci 61:40–53. 10.1016/j.ejps.2014.04.008 24751672 10.1016/j.ejps.2014.04.008

[39] Marasanapalle VP, Crison JR, Ma J et al (2009) Investigation of some factors contributing to negative food effects. Biopharm Drug Dispos 30:71–80. 10.1002/bdd.647 19226652 10.1002/bdd.647

[40] Kasim NA, Whitehouse M, Ramachandran C et al (2004) Molecular properties of WHO essential drugs and provisional biopharmaceutical classification. Mol Pharm 1:85–96. 10.1021/mp034006h 15832504 10.1021/mp034006h

[41] Lilja JJ, Laitinen K, Neuvonen PJ (2005) Effects of grapefruit juice on the absorption of levothyroxine. Br J Clin Pharmacol 60:337–341. 10.1111/j.1365-2125.2005.02433.x 16120075 10.1111/j.1365-2125.2005.02433.x PMC1884777

[42] Singh N, Singh PN, Hershman JM (2000) Effect of calcium carbonate on the absorption of levothyroxine. JAMA 283:2822–2825. 10.1001/jama.283.21.2822 10838651 10.1001/jama.283.21.2822

[43] Centanni M (2013) Thyroxine treatment: absorption, malabsorption, and novel therapeutic approaches. Endocrine 43:8–9. 10.1007/s12020-012-9814-9 23097095 10.1007/s12020-012-9814-9

[44] Hays MT (1968) Absorption of oral thyroxine in man. J Clin Endocrinol Metab 28:749–756. 10.1210/jcem-28-6-749 5656433 10.1210/jcem-28-6-749

[45] Sekadde CB, Slaunwhite WR, Aceto T et al (1974) Administration of thyroxin once a week. J Clin Endocrinol Metab 39:759–764. 10.1210/jcem-39-4-759 4411877 10.1210/jcem-39-4-759

[46] Shahid Mohammed S, Viswaganga Pranush K, Venkatarajagopal Reddy G (2013) Improvement of solubility of omeprazole magnesium by solid dispersion and slugging method. Asian J Res Biol Pharm Sci 1:83–89

[47] Zaid AN, Ghanem M, Maqboul L et al (2016) Biowaiver Eligibility of a Lower Strength Ramipril/Hydrochlorothiazide Immediate Release Tablets Using a New Validated HPLC Analytical Method. Drug Res (Stuttg) 66:539–546. 10.1055/s-0042-111434 27463032 10.1055/s-0042-111434

[48] Meisel S, Shamiss A, Rosenthal T (1994) Clinical pharmacokinetics of ramipril. Clin Pharmacokinet 26:7–15. 10.2165/00003088-199426010-00002 8137599 10.2165/00003088-199426010-00002

[49] Bruchhausen Fv (1994) Hagers handbuch der pharmazeutischen praxis. Stoffe P-Z, 5th ed. Springer Berlin / Heidelberg, Berlin, Heidelberg

[50] Song JC, White CM (2002) Clinical pharmacokinetics and selective pharmacodynamics of new angiotensin converting enzyme inhibitors: an update. Clin Pharmacokinet 41:207–224. 10.2165/00003088-200241030-00005 11929321 10.2165/00003088-200241030-00005

[51] van Griensven JM, Schoemaker RC, Cohen AF et al (1995) Pharmacokinetics, pharmacodynamics and bioavailability of the ACE inhibitor ramipril. Eur J Clin Pharmacol 47:513–518. 10.1007/bf00193704 7768254 10.1007/BF00193704

[52] Rao M, Mandage Y, Thanki K et al. (2010) Dissolution improvement of simvastatin by surface solid dispersion technology. Dissolution Technol 17:27–34. 10.14227/DT170210P27

[53] Lennernäs H, Fager G (1997) Pharmacodynamics and pharmacokinetics of the HMG-CoA reductase inhibitors similarities and differences. Clin Pharmacokinet 32:403–425. 10.2165/00003088-199732050-00005 9160173 10.2165/00003088-199732050-00005

[54] Khan MZI, Rausl D, Radosević S et al (2006) Classification of torasemide based on the Biopharmaceutics Classification System and evaluation of the FDA biowaiver provision for generic products of CLASS I drugs. J Pharm Pharmacol 58:1475–1482. 10.1211/jpp.58.11.0008 17132210 10.1211/jpp.58.11.0008

[55] Fowler SF, Murray KM (1995) Torsemide: a new loop diuretic. Am J Health Syst Pharm 52:1771-80; quiz 1814-5. 10.1093/ajhp/52.16.1771 10.1093/ajhp/52.16.1771 8528833

[56] Knauf H, Mutschler E (1998) Clinical pharmacokinetics and pharmacodynamics of torasemide. Clin Pharmacokinet 34:1–24. 10.2165/00003088-199834010-00001 9474471 10.2165/00003088-199834010-00001

[57] Daescu M, Iota M, Serbschi C et al. (2021) The influence of UV light on photodegradation of acetylsalicylic acid. Int J Mol Sci 22. 10.3390/ijms22084046 10.3390/ijms22084046 PMC8070936 33919943

[58] Menard K, Brostow W, Menard N (2011) Photodegradation of pharmaceuticals studied with UV irradiation and differential scanning calorimetry. Chem Chem Technol 5:385–388

[59] Bakar SK, Niazi S (1983) Stability of aspirin in different media. J Pharm Sci 72:1024–1026. 10.1002/jps.2600720914 6631686 10.1002/jps.2600720914

[60] Blaug SM, Wesolowksi JW (1959) The stability of acetylsalicylic acid in suspension. J Am Pharm Assoc Am Pharm Assoc 48:691–694. 10.1002/jps.3030481205 13849075 10.1002/jps.3030481205

[61] Onah JO (2004) The kinetics of hydrolysis of acetylsalicylic acid (Aspirin) in different polar media. GJPAST 10:297–300. 10.4314/gjpas.v10i2.16397

[62] James KC (1958) The hydrolysis of acetylsalicylic acid from aqueous suspension. J Pharm Pharmacol 10:363–369. 10.1111/j.2042-7158.1958.tb10316.x 13550076 10.1111/j.2042-7158.1958.tb10316.x

[63] Sher M, Iqbal MS, Hussain MA (2013) Comparative hydrolysis study of acetylsalicylic acid and copper - acetylsalicylate by rp-hplc method. J Chem Soc Pak 35:1459–1464

[64] Kornblum SS, Zoglio MA (1967) Pharmaceutical heterogeneous systems. I. Hydrolysis of aspirin in combination with tablet lubricants in an aqueous suspension. J Pharm Sci 56:1569–1575. 10.1002/jps.2600561208 5588707 10.1002/jps.2600561208

[65] Kim HK, Autian J (1960) Binding of Drugs by Plastics II**Received September 10, 1959, from the College of Pharmacy, University of Michigan, Ann Arbor. J Am Pharm Assoc (Scientific ed.) 49:227–230. 10.1002/jps.3030490412

[66] Koch PA, Schultz CA, Wills RJ et al (1978) Influence of food and fluid ingestion on aspirin bioavailability. J Pharm Sci 67:1533–1535. 10.1002/jps.2600671110 712586 10.1002/jps.2600671110

[67] Moore RA, Derry S, Wiffen PJ et al (2015) Effects of food on pharmacokinetics of immediate release oral formulations of aspirin, dipyrone, paracetamol and NSAIDs - a systematic review. Br J Clin Pharmacol 80:381–388. 10.1111/bcp.12628 25784216 10.1111/bcp.12628 PMC4574824

[68] Volans GN (1974) Effects of food and exercise on the absorption of effervescent aspirin. Br J Clin Pharmacol 1:137–141. 10.1111/j.1365-2125.1974.tb00222.x 22454900 10.1111/j.1365-2125.1974.tb00222.x PMC1402447

[69] Ragno G, Cione E, Garofalo A et al (2003) Design and monitoring of photostability systems for amlodipine dosage forms. Int J Pharm 265:125–132. 10.1016/j.ijpharm.2003.07.001 14522125 10.1016/j.ijpharm.2003.07.001

[70] Kawabe Y, Nakamura H, Hino E et al (2008) Photochemical stabilities of some dihydropyridine calcium-channel blockers in powdered pharmaceutical tablets. J Pharm Biomed Anal 47:618–624. 10.1016/j.jpba.2008.01.042 18339506 10.1016/j.jpba.2008.01.042

[71] Ragno G, Garofalo A, Vetuschi C (2002) Photodegradation monitoring of amlodipine by derivative spectrophotometry. J Pharm Biomed Anal 27:19–24. 10.1016/s0731-7085(01)00556-8 11682206 10.1016/s0731-7085(01)00556-8

[72] Gul W, Basheer S, Karim F et al (2015) Effect of acid, base, temperature and UV light on amlodipine besylate. Int J Adv Res Chem Sci 2:21–24

[73] Fasani E, Albini A, Gemme S (2008) Mechanism of the photochemical degradation of amlodipine. Int J Pharm 352:197–201. 10.1016/j.ijpharm.2007.10.040 18068318 10.1016/j.ijpharm.2007.10.040

[74] Stoiljkovic Z, Jadranin M, Djuric S et al (2014) Investigation of forced and total degradation products of amlodipine besylate by liquid chromatography and liquid chromatography-mass spectrometry. CI&CEQ 20:295–304. 10.2298/CICEQ121226011S

[75] Jakimska A, Śliwka-Kaszyńska M, Nagórski P et al (2014) Phototransformation of amlodipine: degradation kinetics and identification of its photoproducts. PLoS ONE 9. 10.1371/journal.pone.0109206 25279815 10.1371/journal.pone.0109206 PMC4184881

[76] Saxena D, Damale S, Joshi A et al (2014) Forced degradation studies of amlodipine besylate and characterization of its major degradation products by LC-MS/MS. Int J Life Sci Biotechnol Pharma Res 3:196–207

[77] Kasagić Vujanović I, Jelić D, Antunović V et al. (2014) Stability study of amlodipine besylate and bisoprolol fumarate in aqueous solutions. CM 5. 10.7251/COMEN1402212V

[78] Chung M, Garza D, Gaffney M et al (2005) Bioavailability of amlodipine besylate following oral administration as a tablet dispersed in applesauce. J Clin Pharmacol 45:695–698. 10.1177/0091270005276203 15901752 10.1177/0091270005276203

[79] Sunkara G, Jiang X, Reynolds C et al (2014) Effect of food on the oral bioavailability of amlodipine/valsartan and amlodipine/valsartan/hydrochlorothiazide fixed dose combination tablets in healthy subjects. Clin Pharmacol Drug Dev 3:487–492. 10.1002/cpdd.131 27129123 10.1002/cpdd.131

[80] Chung M, Calcagni A, Glue P et al (2006) Effect of food on the bioavailability of amlodipine besylate/atorvastatin calcium combination tablet. J Clin Pharmacol 46:1212–1216. 10.1177/0091270006291097 16988211 10.1177/0091270006291097

[81] Piram A, Salvador A, Verne C et al (2008) Photolysis of beta-blockers in environmental waters. Chemosphere 73:1265–1271. 10.1016/j.chemosphere.2008.07.018 18774584 10.1016/j.chemosphere.2008.07.018

[82] Pandey S, Pandey R, Shukla SS (2022) Spectroscopic Substantiation for the Identification of Degradants by Q-TOF Micromass (ESI-MS) in Bisoprolol Fumarate with an Inventive Validation Approach for Stability Indicating HPLC Method. IJPER 56:272–280. 10.5530/ijper.56.1.32

[83] Kasagić-Vujanović I, Stojanović BJ, Ivanović D (2017) Monitoring of bisoprolol fumarate stability under different stress conditions. In: Badnjevic A (ed) Cmbebih 2017: Proceedings of the International Conference on Medical and Biological Engineering 2017, vol 62. Springer Singapore Pte. Limited, Singapore, pp415–424

[84] Al-Asali AAY, Elhag DE, Alamin A (2017) Stability assessment of bisoprolol fumarate under different stress conditions. Euro J Pharm Med Res 4:98–101

[85] Lazarevska-Todevska E, Piponski M, Stefova M (2022) Forced degradation studies and structural characterization of related substances of bisoprolol fumarate in finished drug product using LC-UV-MS/MS. J Serb Chem Soc 87:1185–1202. 10.2298/JSC220204053L

[86] Petřík J, Heřt J, Řezanka P (2021) Development of methodology for the study of API sensitivity to hydrolytic degradations at different pH conditions in solid-state. Chem Pap 75:5739–5747. 10.1007/s11696-021-01630-x

[87] Szalka M, Rokaszewski E, Kaczmarski K (2013) Kinetics of Hydrolysis of Bisoprolol Hemifumarate in Aqueous Acidic Solutions. Int J Chem Kinet 45:744–754. 10.1002/kin.20809

[88] Rao DVS, Radhakrishnanand P, Suryanarayana MV et al (2007) A Stability-Indicating LC Method for Candesartan Cilexetil. Chroma 66:499–507. 10.1365/s10337-007-0364-x

[89] Mehta S, Shah RP, Priyadarshi R et al (2010) LC and LC-MS/TOF studies on stress degradation behaviour of candesartan cilexetil. J Pharm Biomed Anal 52:345–354. 10.1016/j.jpba.2009.05.006 19505786 10.1016/j.jpba.2009.05.006

[90] Phechkrajang CM, Quynh PTN, Suntornsuk L (2017) Forced Degradation Studies of Candesartan Cilexetil and Hydrochlorothiazide Using a Validated Stability-Indicating HPLC-UV Method. Pharm Chem J 51:416–424. 10.1007/s11094-017-1625-0

[91] Amer AM, Allam AN, Abdallah OY (2018) Comparative Pharmaceutical Evaluation of Candesartan and Candesartan Cilexetil: Physicochemical Properties, In Vitro Dissolution and Ex Vivo In Vivo Studies. AAPS PharmSciTech 19:661–667. 10.1208/s12249-017-0879-x 28948575 10.1208/s12249-017-0879-x

[92] Hoppe K, Sznitowska M (2014) The effect of polysorbate 20 on solubility and stability of candesartan cilexetil in dissolution media. AAPS PharmSciTech 15:1116–1125. 10.1208/s12249-014-0109-8 24871550 10.1208/s12249-014-0109-8 PMC4179655

[93] Elhag DE, Riad MNE, Ahmed SA (2016) RP-HPLC hydrolytic stability study of candesartan cilexetil. World J Pharm Res 5:321–332

[94] Rajput RS, Lariya N (2022) A stability indicating method development and validation of esomeprazole in pharmaceutical dosage form by using RP-HPLC and In Vitro evaluation of nasogastric tube delivery of esomeprazole magnesium delayed-release capsules. J Med Pharm Allied Sci 11:4375–4381

[95] Gul W, Sajid S, Hamid F et al (2015) Effect of acidic Ph. and heat on the degradation of omeprazole and esomeprazole. Pharma Innov J 4(8, Part A):19–21

[96] Chen F, He X, Fang B et al (2020) Simultaneous Quantitative Analysis of Six Proton-Pump Inhibitors with a Single Marker and Evaluation of Stability of Investigated Drugs in Polypropylene Syringes for Continuous Infusion Use. Drug Des Devel Ther 14:5689–5698. 10.2147/DDDT.S279302 33380789 10.2147/DDDT.S279302 PMC7769080

[97] Bladh N, Blychert E, Johansson K et al (2007) A new esomeprazole packet (sachet) formulation for suspension: in vitro characteristics and comparative pharmacokinetics versus intact capsules/tablets in healthy volunteers. Clin Ther 29:640–649. 10.1016/j.clinthera.2007.03.014 17617287 10.1016/j.clinthera.2007.03.014

[98] Johnson DA, Roach AC, Carlsson AS et al (2003) Stability of esomeprazole capsule contents after in vitro suspension in common soft foods and beverages. Pharmacotherapy 23:731–734. 10.1592/phco.23.6.731.32181 12820815 10.1592/phco.23.6.731.32181

[99] Kupiec TC, Aloumanis V, Ben M et al (2008) Physical and chemical stability of esomeprazole sodium solutions. Ann Pharmacother 42:1247–1251. 10.1345/aph.1L079 18614750 10.1345/aph.1L079

[100] Hoover A, Sun D, Wen H et al (2017) In Vitro Evaluation of Nasogastric Tube Delivery Performance of Esomeprazole Magnesium Delayed-Release Capsules. J Pharm Sci 106:1859–1864. 10.1016/j.xphs.2017.04.008 28416417 10.1016/j.xphs.2017.04.008 PMC5564165

[101] Stewart P, Dayneka N, Grenier S et al (2009) In vitro study of esomeprazole sachet suspension administered via enteral feeding tubes. Can J Hosp Pharm 62:48–49. 10.4212/cjhp.v62i1.122 22478867 10.4212/cjhp.v62i1.122 PMC2826898

[102] Shah SA, Sander S, Coleman CI et al (2006) Delivery of esomeprazole magnesium through nasogastric and gastrostomy tubes using an oral liquid vehicle as a suspending agent in vitro. Am J Health Syst Pharm 63:1882–1887. 10.2146/ajhp060025 16990636 10.2146/ajhp060025

[103] Sostek MB, Chen Y, Skammer W et al (2003) Esomeprazole administered through a nasogastric tube provides bioavailability similar to oral dosing. Aliment Pharmacol Ther 18:581–586. 10.1046/j.1365-2036.2003.01667.x 12969084 10.1046/j.1365-2036.2003.01667.x

[104] Shi S, Klotz U (2008) Proton pump inhibitors: an update of their clinical use and pharmacokinetics. Eur J Clin Pharmacol 64:935–951. 10.1007/s00228-008-0538-y 18679668 10.1007/s00228-008-0538-y

[105] Sostek MB, Chen Y, Andersson T (2007) Effect of timing of dosing in relation to food intake on the pharmacokinetics of esomeprazole. Br J Clin Pharmacol 64:386–390. 10.1111/j.1365-2125.2007.02889.x 17425628 10.1111/j.1365-2125.2007.02889.x PMC2000656

[106] Carda-Broch S, Esteve-Romero J, García-Alvarez-Coque MC (2000) Furosemide assay in pharmaceuticals by Micellar liquid chromatography: study of the stability of the drug. J Pharm Biomed Anal 23:803–817. 10.1016/s0731-7085(00)00378-2 11022906 10.1016/s0731-7085(00)00378-2

[107] Bundgaard H, Nørgaard T, Nielsen NM (1988) Photodegradation and hydrolysis of furosemide and furosemide esters in aqueous solutions. Int J Pharm 42:217–224. 10.1016/0378-5173(88)90178-0

[108] Yagi N, Kenmotsu H, Sekikawa H et al (1991) Studies on the photolysis and hydrolysis of furosemide in aqueous solution. Chem Pharm Bull 39:454–457. 10.1248/cpb.39.454

[109] Cies JJ, Moore WS, Chopra A et al (2015) Stability of furosemide and chlorothiazide stored in syringes. Am J Health Syst Pharm 72:2182–2188. 10.2146/ajhp150023 26637518 10.2146/ajhp150023 PMC4834703

[110] Asker AF, Ferdous AJ (1996) Photodegradation of furosemide solutions. PDA J Pharm Sci Technol 50:158–162 8696779

[111] Chen F, Fang B, Wang S (2021) A Fast and Validated HPLC Method for Simultaneous Determination of Dopamine, Dobutamine, Phentolamine, Furosemide, and Aminophylline in Infusion Samples and Injection Formulations. J Anal Methods Chem 2021:8821126. 10.1155/2021/8821126 33728093 10.1155/2021/8821126 PMC7936887

[112] Cruz JE, Maness DD, Yakatan GJ (1979) Kinetics and mechanism of hydrolysis of furosemide. Int J Pharm 2:275–281. 10.1016/0378-5173(79)90034-6

[113] Ghanekar AG, Das Gupta V, Gibbs CW (1978) Stability of furosemide in aqueous systems. J Pharm Sci 67:808–811. 10.1002/jps.2600670621 660463 10.1002/jps.2600670621

[114] Shah KA, Das Gupta V, Stewart KR (1980) Effect of pH, chlorobutanol, cysteine hydrochloride, ethylenediaminetetraacetic acid, propylene glycol, sodium metabisulfite, and sodium sulfite on furosemide stability in aqueous solutions. J Pharm Sci 69:594–596. 10.1002/jps.2600690533 6770073 10.1002/jps.2600690533

[115] van der Schaar JAJ, Grouls R, Franssen EJF et al (2019) Stability of Furosemide 5 mg/mL in Polypropylene Syringes. Int J Pharm Compd 23:414–417 31513540

[116] Chentoufi MA, Bennis S, Benabbes M et al. (2018) 3PC-009 Physicochemical stability of intravenous injection of a generic product of furosemide prepared in polypropylene syringes:A27.2-A28. 10.1136/ejhpharm-2018-eahpconf.61

[117] Donnelly RF (2002) Chemical stability of furosemide in minibags and polypropylene syringes. Int J Pharm Compd 6:468–470 23979472

[118] Michael RK, Ralph EC, Arden WF et al (1974) Pharmacokinetics of orally administered furosemide. Clin Pharmacol Ther 15:178–186. 10.1002/cpt1974152178 4812154

[119] Hammarlund MM, Paalzow LK, Odlind B (1984) Pharmacokinetics of furosemide in man after intravenous and oral administration. Application of moment analysis. Eur J Clin Pharmacol 26:197–207. 10.1007/BF00630286 6723758 10.1007/BF00630286

[120] Beermann B, Midskov C (1986) Reduced bioavailability and effect of furosemide given with food. Eur J Clin Pharmacol 29:725–727. 10.1007/bf00615967 3709617 10.1007/BF00615967

[121] McCrindle JL, Li Kam Wa TC, Barron W et al (1996) Effect of food on the absorption of frusemide and bumetanide in man. Br J Clin Pharmacol 42:743–746. 10.1046/j.1365-2125.1996.00494.x 8971430 10.1046/j.1365-2125.1996.00494.x PMC2042717

[122] Ogata H, Kawatsu Y, Maruyama Y et al (1985) Bioavailability and diuretic effect of furosemide during long-term treatment of chronic respiratory failure. Eur J Clin Pharmacol 28:53–59. 10.1007/bf00635708 3987786 10.1007/BF00635708

[123] Bard RL, Bleske BE, Nicklas JM (2004) Food: an unrecognized source of loop diuretic resistance. Pharmacotherapy 24:630–637. 10.1592/phco.24.6.630.34736 15162897 10.1592/phco.24.6.630.34736

[124] Mahajan AA, Thaker AK, Mohanraj K (2012) LC, LC-MS/MS studies for the identification and characterization of degradation products of hydrochlorothiazide and establishment of mechanistic approach towards degradation. J Braz Chem Soc. 10.1590/S0103-50532012000300010

[125] Kamble RM, Singh S, Singh S (2010) Development and validation of a stability indicating LC method for the determination of hydrochlorothiazide in pharmaceutical formulations. J Pharm Res 3:2949–2952

[126] Deventer K, Baele G, van Eenoo P et al (2009) Stability of selected chlorinated thiazide diuretics. J Pharm Biomed Anal 49:519–524. 10.1016/j.jpba.2008.11.001 19108977 10.1016/j.jpba.2008.11.001

[127] Barbhaiya RH, Craig WA, Corrick-West HP et al (1982) Pharmacokinetics of hydrochlorothiazide in fasted and nonfasted subjects: a comparison of plasma level and urinary excretion methods. J Pharm Sci 71:245–248. 10.1002/jps.2600710226 7062255 10.1002/jps.2600710226

[128] Beermann B, Groschinsky-Grind M (1978) Gastrointestinal absorption of hydrochlorothiazide enhanced by concomitant intake of food. Eur J Clin Pharmacol 13:125–128. 10.1007/bf00609756 658108 10.1007/BF00609756

[129] Pappert EJ, Buhrfiend C, Lipton JW et al (1996) Levodopa stability in solution: time course, environmental effects, and practical recommendations for clinical use. Mov Disord 11:24–26. 10.1002/mds.870110106 8771063 10.1002/mds.870110106

[130] Pereira RL, Paim CS, Barth AB et al (2012) Levodopa microparticles for pulmonary delivery: photodegradation kinetics and LC stability-indicating method. Pharmazie 67:605–610 22888517

[131] Zhou YZ, Alany RG, Chuang V et al (2012) Studies of the Rate Constant of l-DOPA Oxidation and Decarboxylation by HPLC. Chroma 75:597–606. 10.1007/s10337-012-2229-1

[132] Weitzel J, Wünsch A, Rose O et al (2022) Different dissolution conditions affect stability and dissolution profiles of bioequivalent levodopa-containing oral dosage forms. Int J Pharm 629. 10.1016/j.ijpharm.2022.122401 36395922 10.1016/j.ijpharm.2022.122401

[133] Pincus JH, Barry KM (1987) Plasma levels of amino acids correlate with motor fluctuations in parkinsonism. Arch Neurol 44:1006–1009. 10.1001/archneur.1987.00520220012007 3632370 10.1001/archneur.1987.00520220012007

[134] Robertson DR, Higginson I, Macklin BS et al (1991) The influence of protein containing meals on the pharmacokinetics of levodopa in healthy volunteers. Br J Clin Pharmacol 31:413–417. 10.1111/j.1365-2125.1991.tb05555.x 2049250 10.1111/j.1365-2125.1991.tb05555.x PMC1368327

[135] Simon N, Gantcheva R, Bruguerolle B et al (2004) The effects of a normal protein diet on levodopa plasma kinetics in advanced Parkinson's disease. Parkinsonism Relat Disord 10:137–142. 10.1016/j.parkreldis.2003.10.004 15036167 10.1016/j.parkreldis.2003.10.004

[136] Bonnici A, Ruiner C-E, St-Laurent L et al (2010) An interaction between levodopa and enteral nutrition resulting in neuroleptic malignant-like syndrome and prolonged ICU stay. Ann Pharmacother 44:1504–1507. 10.1345/aph.1P242 20628041 10.1345/aph.1P242

[137] Cooper MK, Brock DG, McDaniel CM (2008) Interaction between levodopa and enteral nutrition. Ann Pharmacother 42:439–442. 10.1345/aph.1K450 18272698 10.1345/aph.1K450

[138] Parizi MPS, Lastre Acosta AM, Ishiki HM et al (2019) Environmental photochemical fate and UVC degradation of sodium levothyroxine in aqueous medium. Environ Sci Pollut Res Int 26:4393–4403. 10.1007/s11356-018-2907-0 30109685 10.1007/s11356-018-2907-0

[139] Abdallah S, Mohamed I (2016) Factor Affecting Photo and Thermal Stability of Levothyroxine Sodium. BJPR 10:1–11. 10.9734/BJPR/2016/23410

[140] Svanfelt J, Eriksson J, Kronberg L (2011) Photochemical transformation of the thyroid hormone levothyroxine in aqueous solution. Environ Sci Pollut Res Int 18:871–876. 10.1007/s11356-011-0450-3 21274638 10.1007/s11356-011-0450-3

[141] Frenette AJ, MacLean RD, Williamson D et al (2011) Stability of levothyroxine injection in glass, polyvinyl chloride, and polyolefin containers. Am J Health Syst Pharm 68:1723–1728. 10.2146/ajhp100599 21880888 10.2146/ajhp100599

[142] Won CM (1992) Kinetics of degradation of levothyroxine in aqueous solution and in solid state. Pharm Res 9:131–137. 10.1023/A:1018952415732 1589398 10.1023/a:1018952415732

[143] Stadalman KA, Kelner MJ, Box K et al. (2009) Stability of levothyroxine sodium 0.4 microg/mL in 0.9% sodium chloride injection. Prog Transplant 19:354-6; quiz 357. 10.1177/152692480901900411 10.1177/152692480901900411 20050459

[144] Gupta VD (2000) Stability of levothyroxine sodium injection in polypropylene syringes. Int J Pharm Compd 4:482–483 23981741

[145] Strong DK, Decarie D, Ensom MHH (2010) Stability of Levothyroxine in Sodium Chloride for IV Administration. Can J Hosp Pharm 63:437–443. 10.4212/cjhp.v63i6.963 22479016 10.4212/cjhp.v63i6.963 PMC3004701

[146] Svirskis D, Lin S-W, Brown H et al (2018) The Influence of Tablet Formulation, Drug Concentration, and pH Modification on the Stability of Extemporaneously Compounded Levothyroxine Suspensions. Int J Pharm Compd 22:164–171 29877863

[147] Golombek SG, Alpan G, Frey M et al (2011) Stability of thyroid hormones during continuous infusion. J Perinat Med 39:471–475. 10.1515/JPM.2011.051 21501101 10.1515/jpm.2011.051

[148] Pirola I, Daffini L, Gandossi E et al (2014) Comparison between liquid and tablet levothyroxine formulations in patients treated through enteral feeding tube. J Endocrinol Invest 37:583–587. 10.1007/s40618-014-0082-9 24789541 10.1007/s40618-014-0082-9

[149] Manessis A, Lascher S, Bukberg P et al (2008) Quantifying amount of adsorption of levothyroxine by percutaneous endoscopic gastrostomy tubes. JPEN J Parenter Enteral Nutr 32:197–200. 10.1177/0148607108314770 18407914 10.1177/0148607108314770

[150] Campbell NR, Hasinoff BB, Stalts H et al (1992) Ferrous sulfate reduces thyroxine efficacy in patients with hypothyroidism. Ann Intern Med 117:1010–1013. 10.7326/0003-4819-117-12-1010 1443969 10.7326/0003-4819-117-12-1010

[151] Zamfirescu I, Carlson HE (2011) Absorption of levothyroxine when coadministered with various calcium formulations. Thyroid 21:483–486. 10.1089/thy.2010.0296 21595516 10.1089/thy.2010.0296 PMC3092723

[152] Jabbar MA, Larrea J, Shaw RA (1997) Abnormal thyroid function tests in infants with congenital hypothyroidism: the influence of soy-based formula. J Am Coll Nutr 16:280–282. 10.1080/07315724.1997.10718686 9176836 10.1080/07315724.1997.10718686

[153] Bell DS, Ovalle F (2001) Use of soy protein supplement and resultant need for increased dose of levothyroxine. Endocr Pract 7:193–194. 10.4158/EP.7.3.193 11421567 10.4158/EP.7.3.193

[154] Liel Y, Harman-Boehm I, Shany S (1996) Evidence for a clinically important adverse effect of fiber-enriched diet on the bioavailability of levothyroxine in adult hypothyroid patients. J Clin Endocrinol Metab 81:857–859. 10.1210/jcem.81.2.8636317 8636317 10.1210/jcem.81.2.8636317

[155] Dickerson RN, Maish GO, Minard G et al (2010) Clinical relevancy of the levothyroxine-continuous enteral nutrition interaction. Nutr Clin Pract 25:646–652. 10.1177/0884533610385701 21139130 10.1177/0884533610385701

[156] Shankar G, Borkar RM, Udutha S et al (2019) Identification and structural characterization of the stress degradation products of omeprazole using Q-TOF-LC-ESI-MS/MS and NMR experiments: evaluation of the toxicity of the degradation products. New J Chem 43:7294–7306. 10.1039/c9nj00932a

[157] Ruiz MA, Reyes I, Parera A et al (1998) Determination of the stability of omeprazole by means of differential scanning calorimetry. J Therm Anal 51:29–35

[158] Jackson R, Lewis P, Brown SD (2020) Comparative Stability of Compounded Omeprazole Suspension Versus Commercial Omeprazole Kit When Stored in Oral Syringes Under Refrigerated Conditions. J Pharm Technol 36:179–186. 10.1177/8755122520935532 34752549 10.1177/8755122520935532 PMC7453476

[159] Mathew M, Gupta VD, Bailey RE (1995) Stability of Omeprazole Solutions at Various ph Values as Determined by High-Performance Liquid Chromatography. Drug Dev Ind Pharm 21:965–971. 10.3109/03639049509026660

[160] El-Badry M, Taha AI, Alanazi FK et al (2009) Study of omeprazole stability in aqueous solution: inluence of cyclodextrins. J Drug Deliv Sci Technol 19:347–351

[161] Ekpe A, Jacobsen T (1999) Effect of various salts on the stability of lansoprazole, omeprazole, and pantoprazole as determined by high-performance liquid chromatography. Drug Dev Ind Pharm 25:1057–1065. 10.1081/DDC-100102270 10518247 10.1081/ddc-100102270

[162] DiGiacinto JL, Olsen KM, Bergman KL et al (2000) Stability of Suspension Formulations of Stability of Suspension Formulations of Lansoprazole and Omeprazole Stored in Amber-Colored Plastic Oral Syringes. Ann Pharmacother 34:600–605 10852086 10.1345/aph.19086

[163] Johnson CE, Cober MP, Ludwig JL (2007) Stability of partial doses of omeprazole-sodium bicarbonate oral suspension. Ann Pharmacother 41:1954–1961. 10.1345/aph.1K246 17956960 10.1345/aph.1K246

[164] Baniasadi S, Kobarfard F, Fahimi F (2012) Extemporaneous preparation and stability assessment of omeprazole suspension in a teaching hospital. Int J Pharmacy Teach Prac 3:418–421

[165] Burnett JE, Balkin ER (2006) Stability and viscosity of a flavored omeprazole oral suspension for pediatric use. Am J Health Syst Pharm 63:2240–2247. 10.2146/ajhp060026 17090745 10.2146/ajhp060026

[166] Garg S, Svirskis D, Al-Kabban M et al (2009) Chemical stability of extemporaneously compounded omeprazole formulations: a comparison of two methods of compounding. Int J Pharm Compd 13:250–253 23966480

[167] Quercia RA, Fan C, Liu X et al (1997) Stability of omeprazole in an extemporaneously prepared oral liquid. Am J Health Syst Pharm 54:1833–1836 9269520 10.1093/ajhp/54.16.1833

[168] Carpenter JF, McNulty MA, Dusci LJ et al (2006) Stability of omeprazole sodium and pantoprazole sodium diluted for intravenous infusion. J Pharmacy Technol 22:95–98

[169] Meissner S, Bansal M, Dela CPD et al (2020) The Effect of Manufacturer on the Compounding of Omeprazole Suspensions and Their Stability Assessment. Int J Pharm Compd 24:140–147 32196476

[170] Thomson AB, Sinclair P, Matisko A et al (1997) Influence of food on the bioavailability of an enteric-coated tablet formulation of omeprazole 20 mg under repeated dose conditions. Can J Gastroenterol 11:663–667. 10.1155/1997/830856 9459045 10.1155/1997/830856

[171] Pilbrant A, Cederberg C (1985) Development of an oral formulation of omeprazole. Scand J Gastroenterol Suppl 108:113–120. 10.3109/00365528509095824 3858973 10.3109/00365528509095824

[172] de Diego M, Godoy G, Mennickent S et al (2010) Stress degradation studies of ramipril by a validated stability-indicating liquid chromatographic method. J Chil Chem Soc 55(4):450–453

[173] Elshanawane AA, Mostafa SM, Elgawish MS (2008) Application of a Validated, Stability-Indicating LC Method to Stress Degradation Studies of Ramipril and Moexipril.HCl. Chroma 67:567–573. 10.1365/s10337-008-0544-3

[174] Hanysová L, Václavková M, Dohnal J et al (2005) Stability of ramipril in the solvents of different pH. J Pharm Biomed Anal 37:1179–1183. 10.1016/j.jpba.2004.10.041 15862704 10.1016/j.jpba.2004.10.041

[175] Allen LV, Stiles ML, Prince SJ et al (1995) Stability of ramipril in water, apple juice, and applesauce. Am J Health Syst Pharm 52:2433–2436. 10.1093/ajhp/52.21.2433 8564609 10.1093/ajhp/52.21.2433

[176] Mielcarek J, Naskrent M, Grobelny P (2009) Photochemical properties of simvastatin and lovastatin induced by radiation. J Therm Anal Calorim 96:301–305. 10.1007/s10973-008-9322-6

[177] Piecha M, Sarakha M, Trebše P et al (2010) Stability studies of cholesterol lowering statin drugs in aqueous samples using HPLC and LC–MS. Environ Chem Lett 8:185–191. 10.1007/s10311-009-0207-0

[178] Alvarez-Lueje A, Valenzuela C, Squella JA et al (2005) Stability study of simvastatin under hydrolytic conditions assessed by liquid chromatography. J AOAC Int 88:1631–1636 16526443

[179] Malenović A, Jančić-Stojanović B, Ivanović D et al (2010) Forced degradation studies of simvastatin using microemulsion liquid chromatography. J Liq Chromatogr Relat Technol 33:536–547. 10.1080/10826070903574576

[180] Nakai A, Nishikata M, Matsuyama K et al (1996) Drug interaction between simvastatin and cholestyramine in vitro and in vivo. Biol Pharm Bull 19:1231–1233. 10.1248/bpb.19.1231 8889048 10.1248/bpb.19.1231

[181] Garnett WR (1995) Interactions with hydroxymethylglutaryl-coenzyme A reductase inhibitors. Am J Health Syst Pharm 52:1639–1645. 10.1093/ajhp/52.15.1639 7583826 10.1093/ajhp/52.15.1639

[182] Kakde R, Chaudhary N, Barsagade A et al (2011) Stability-indicating HPTLC method for analysis of torsemide in pharmaceutical preparations. Acta Chromatogr 23:145–155. 10.1556/AChrom.23.2011.1.10

[183] Jovic Z, Zivanovic L, Protic A et al (2013) Forced degradation study of torasemide: characterization of its degradation products. J Liq Chromatogr Relat Technol 36:2082–2094. 10.1080/10826076.2012.712932

[184] Montgomery PA, Cornish LA, Johnson CE et al (1998) Stability of torsemide in 5% dextrose injection. Am J Health Syst Pharm 55:1042–1043. 10.1093/ajhp/55.10.1042 9606457 10.1093/ajhp/55.10.1042

[185] Blose JS, Adams KF, Patterson JH (1995) Torsemide: a pyridine-sulfonylurea loop diuretic. Ann Pharmacother 29:396–402. 10.1177/106002809502900411 7633019 10.1177/106002809502900411

[186] Kramer WG (1995) Effect of Food on the Pharmacokinetics and Pharmacodynamics of Torsemide. Am J Ther 2:499–503. 10.1097/00045391-199506000-00010 11850698 10.1097/00045391-199506000-00010

[187] Amidon GL, Lennernäs H, Shah VP et al (1995) A theoretical basis for a biopharmaceutic drug classification: the correlation of in vitro drug product dissolution and in vivo bioavailability. Pharm Res 12:413–420. 10.1023/a:1016212804288 7617530 10.1023/a:1016212804288

[188] U.S. Department of Health and Human Services Food and Drug Administration Center for Drug Evaluation and Research (2017) Waiver of in vivo bioavailability and bioequivalence studies for immediate-release solid oral dosage forms based on a biopharmaceutics classification system: Guidance for industry. https://collections.nlm.nih.gov/catalog/nlm:nlmuid-101720038-pdf. Accessed 11 Jun 2024

[189] Food And Drug Administration (2021) Oral drug products administered via enteral feeding tube: in vitro testing and labelling recommendations: guidance for industry. https://www.fda.gov/media/149688/download. Accessed 27 Mar 2024

[190] Karkossa F, Lehmann N, Klein S (2022) A systematic approach for assessing the suitability of enteral feeding tubes for the administration of controlled-release pellet formulations. Int J Pharm 612. 10.1016/j.ijpharm.2021.121286 34775043 10.1016/j.ijpharm.2021.121286

[191] Karkossa F, Bading A, Klein S (2024) What to consider for successful administration of oral liquids via enteral feeding tubes? a case study with paediatric ibuprofen suspensions. Int J Pharm 649. 10.1016/j.ijpharm.2023.123628 37984617 10.1016/j.ijpharm.2023.123628

[192] Kumarathunga PADM, Kalupahana NS, Antonypillai CN (2021) Over-the-counter protein supplement resulting in impaired thyroxine absorption in a hypothyroid patient. Endocrinol Diabetes Metab Case Rep 2021. 10.1530/EDM-21-0070 10.1530/EDM-21-0070 PMC8346176 34280893

[193] Anderton A (1984) Scanning electron microscopy of the internal wall topography of enteral feeding tubes. Clin Nutr 3:171–174. 10.1016/S0261-5614(84)80040-0R 16829456

