# Supplementary material for: Drug administration via feeding tubes—a procedure that carries risks: systematic identification of critical factors based on commonly administered drugs in a cohort of stroke patients
Source: Eur J Clin Pharmacol. 2024 Jul 29;80(11):1599–623. doi: 10.1007/s00228-024-03723-4 (PMC11458809; doi:10.1007/s00228-024-03723-4)
Supplement: Supplementary file 1 — Supplementary file1 (DOCX 23 KB) [file 228_2024_3723_MOESM1_ESM.docx]

Supplementary material

**Drug administration via feeding tubes - a procedure that carries risks: Systematic identification of critical factors based on commonly administered drugs in a cohort of stroke patients**

Table 1 shows the search terms and the corresponding number of search results of the first literature search. Some search strategies provided a high number of search results (written in bold). These were narrowed down by additional search terms (written in italics).

**Tab 1** Search terms and number of results of the initial literature search for the most commonly used drugs according to the letters of discharge; dn – drug name: 1 acetylsalicylic acid, 2 amlodipine, 3 bisoprolol, 4 candesartan, 5 esomeprazole, 6 furosemide, 7 hydrochlorothiazide, 8 omeprazole, 9 ramipril, 10 simvastatin, 11 torasemide, 12 levodopa, 13 levothyroxine sodium.

| **Search terms** | **1** | **2** | **3** | **4** | **5** | **6** | **7** | **8** | **9** | **10** | **11** | **12** | **13** |
| --- | --- | --- | --- | --- | --- | --- | --- | --- | --- | --- | --- | --- | --- |
| dn AND interaction AND food | 64 | 1 | 1 | 5 | 7 | 12 | 10 | 1 | 1 | 25 | 1 | 18 | 101 |
| dn AND food-drug-interaction | 165 | 21 | 4 | 5 | 13 | 20 | 20 | 70 | 6 | 64 | 0 | 26 | 48 |
| **dn AND pharmacokinetics** | **2100** | **401** | 123 | 164 | 191 | **1280** | **537** | **1385** | 144 | **640** | 97 | 123 | 84 |
| *dn AND pharmacokinetics AND human AND oral* | *127* | *58* |  |  |  | *1* | *46* | *176* |  | *73* |  |  |  |
| **dn AND solubility** | ***390*** | 30 | 14 | 24 | 18 | 134 | 114 | 71 | 17 | 114 | 3 | 63 | 107 |
| *dn AND solubility AND human AND oral* | *30* |  |  |  |  |  |  |  |  |  |  |  |  |
| dn AND pka AND solubility | 2 | 1 |  | 1 | 2 | 4 | 2 |  |  |  |  |  |  |
| dn AND pharmacokinetics AND pka | 5 | 3 |  |  | 1 |  | 1 | 8 |  |  |  |  |  |
| dn AND fasted AND fed | 15 | 1 |  |  | 3 | 11 | 5 | 26 | 1 | 3 |  | 5 | 144 |
| dn AND fasted AND food | 10 | 2 |  |  | 4 | 8 | 3 | 20 | 1 |  |  | 7 | 93 |
| dn AND feeding tubes |  |  |  |  | 2 | 1 |  | 5 |  | 2 |  | 1 | 2 |
| dn AND enteral feed | 9 | 1 | 1 |  |  | 14 |  | 13 |  | 3 |  | 9 | 30 |
| Publications selected for further evaluation | 3 | 7 | 2 | 4 | 3 | 7 | 4 | 3 | 3 | 2 | 5 | 5 | 11 |

The second literature review was conducted in PubMed and Google Scholar and focused on the stability of the selected drug substances in the context of administration via feeding tubes. Photolysis, hydrolysis and oxidation sensitivity were additional interests. The search terms and the corresponding numbers of results are listed in table 2.

**Tab 2** Search terms and number of results of the second literature search for the most commonly used rugs based on evaluation of the letters of discharge; first row results from PubMed, second row results from Google Scholar;
dn: 1 acetylsalicylic acid, 2 amlodipine, 3 bisoprolol, 4 candesartan, 5 esomeprazole, 6 furosemide, 7 hydrochlorothiazide, 8 omeprazole, 9 ramipril, 10 simvastatin, 11 torasemide, 12 levodopa,
13 levothyroxine sodium

| **Search terms** | **1** | **2** | **3** | **4** | **5** | **6** | **7** | **8** | **9** | **10** | **11** | **12** | **13** |
| --- | --- | --- | --- | --- | --- | --- | --- | --- | --- | --- | --- | --- | --- |
| dn AND stability | 1,113  39,600 | 193  31,500 | 56  7,200 | 83  12,500 | 54  5,930 | 294  29,300 | 211  18,300 | 304  27,300 | 73  22,300 | 409  57,600 | 19  6,930 | 467  28,800 | 797  25,800 |
| dn AND stability AND aqueous | 44  18,100 | 7  16,200 | 1  2,650 | 8  5,400 | 2  2,150 | 15  14,600 | 18  10,200 | 26  15,000 | 5  12,400 | 23  23,900 | 0  1,320 | 16  10,700 | 9  17,600 |
| dn AND stability AND suspension | 22  14,000 | 9  15,300 | 0  1,960 | 4  4,210 | 5  2,260 | 8  13,000 | 10  7,000 | 26  11,700 | 3  10,700 | 10  21,700 | 2  589 | 7  9,300 | 5  18,100 |
| dn AND stability AND water | 98  27,900 | 24  29,400 | 6  7,970 | 15  13,900 | 12  5,830 | 48  32,300 | 50  20,800 | 32  25,600 | 8  19,900 | 42  39,700 | 5  2,490 | 35  25,100 | 32  22,400 |
| dn AND stability AND syringe | 1  5,170 | 0  6,670 | 0  821 | 0  1,110 | 2  781 | 7  6,510 | 0  2,820 | 7  3,730 | 0  3,880 | 0  8,320 | 0  106 | 1  3,280 | 2  16,900 |
| dn AND stability AND photolysis | 4  12,700 | 4  1,150 | 1  440 | 2  287 | 1  206 | 3  1,060 | 6  1,180 | 3  632 | 0  677 | 0  1,020 | 0  91 | 0  1,220 | 1  5,910 |
| dn AND stability AND oxidation | 126  22,400 | 29  22,800 | 5  7,720 | 10  14,500 | 10  4,820 | 26  19,900 | 32  15,600 | 26  19,700 | 7  18,500 | 71  31,900 | 2  2,180 | 48  19,900 | 34  20,000 |
| dn AND stability AND hydrolysis | 68  11,800 | 9  14,000 | 1  258 | 5  5,460 | 2  1,720 | 5  9,800 | 15  5,340 | 8  9,670 | 1  13,500 | 7  20,700 | 1  730 | 16  8,630 | 11  18,000 |
| Number of publications selected for further evaluation | 9 | 9 | 8 | 6 | 10 | 12 | 4 | 14 | 4 | 5 | 3 | 4 | 12 |
